# Niclosamide extends health span and reduces frailty by ameliorating mTORC1 hyperactivation in aging models

**DOI:** 10.1016/j.jare.2025.04.027

**Published:** 2025-04-22

**Authors:** Pyeong Geun Choi, Hee Soo Kim, So-Hyun Park, Hyo-Deok Seo, Jeong-Hoon Hahm, Yang Hoon Huh, Tae-Il Jeon, Jiyun Ahn, Chang Hwa Jung

**Affiliations:** a Aging Research Group, Korea Food Research Institute, Wanju-gun, Republic of Korea; b Department of Food Biotechnology, Korea University of Science and Technology, Wanju-gun, Republic of Korea; c Center for Electron Microscopy Research, Korea Basic Science Institute, Cheongju, Republic of Korea; d Department of Animal Science, Chonnam National University, Gwangju, Republic of Korea

**Keywords:** Aging, *Caenorhabditis elegans*, Health span, Longevity, Sarcopenia, Skeletal muscle

## Abstract

**Introduction:**

Frailty is characterized by an increased vulnerability to disease and physical debilitation due to a decline in the body’s capacity to maintain homeostasis during aging. Therefore, effective management of frailty is crucial for promoting health. Although the role of niclosamide (NIC), an autophagy promoter, has been studied for the treatment of cancer, infectious diseases, and metabolic disorders, no research has focused on its effects on aging.

**Objectives:**

In this study, we aimed to evaluate the effects of NIC on the aging process and assess its potential as a novel anti-aging therapeutic agent.

**Methods:**

We evaluated the effects of NIC on frailty, physical function, and metabolic function using *Caenorhabditis elegans* (*C. elegans*) and aging mouse models. NIC effectiveness was assessed using behavioral experiments, histological analysis, and molecular biological analysis.

**Results:**

We identified NIC as a compound that enhanced exercise capacity and metabolism, thereby alleviating frailty. Briefly, NIC extended the lifespan and improved frailty-related phenotypes in *C. elegans*, and effectively ameliorated frailty in aging mice, particularly in muscle aging. Additionally, NIC treatment suppressed the muscle atrophy-related ubiquitin–proteasome system induced by mammalian target of rapamycin complex 1 (mTORC1) hyperactivation, while enhancing autophagic flux, another aspect of proteostasis. Furthermore, mRNA-seq analysis revealed that NIC improved metabolism-related functions.

**Conclusion:**

Collectively, these findings suggest that NIC is a promising novel candidate for the prevention of frailty.

## Highlights

• Niclosamide (NIC) extended the lifespan and improved frailty-related phenotypes in *C. elegans.*
• NIC effectively ameliorated frailty in aging mice, particularly in muscle aging.
• NIC treatment suppressed mTORC1 hyperactivation by aging.
• NIC treatment enhanced autophagic flux and improved metabolism-related functions.
• Thus, NIC is a promising novel candidate for the prevention of frailty.

## Graphical abstract

**Figure:**
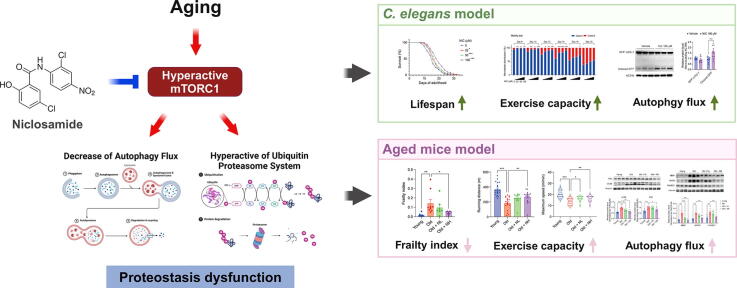

## Introduction

Autophagy involves the breakdown of various molecules and cellular components, such as organelles, lipids, proteins, and nucleic acids, through lysosomal fusion, making it a crucial process for maintaining cellular homeostasis. Autophagy is an evolutionarily conserved pathway in unicellular organisms, such as yeast, and complex multicellular organisms, such as flies, worms, and mammals, and has been linked to aging [1,2]. Numerous studies have suggested that autophagic activity decreases with age in various organisms. Age-dependent decline in lysosomal function impairs the autophagic flux, exacerbates cellular impairment, and contributes to the development of age-related diseases [3]. Moreover, recent studies have suggested that autophagy is involved in longevity. In *Caenorhabditis elegans* (*C. elegans*), the downstream pathways of various longevity models, including reduced TOR signaling, insulin/IGF-1 signaling, dietary restriction, reduced mitochondrial respiration, and germline removal, involve the autophagy pathway, and the loss of function of autophagy-related genes have been shown to abolish the longevity phenotype [4]. In mice, autophagy activation by ATG5 overexpression extended the lifespan [5] and autophagy-promoting compounds, including urolithin A [6], rapamycin [7], and spermidine [8] mitigated the deterioration caused by aging. Recent studies have led to growing interest in identifying autophagy-regulating substances that may promote longevity and help elucidate the mechanisms behind their effects.

Niclosamide (NIC) is a medication that has been included in the World Health Organization’s list of essential medicines since the 1960 s, and is used to treat tapeworm infection [9]. The mechanism of action of NIC involves uncoupling oxidative phosphorylation in the mitochondria, which disrupts the tapeworm’s ability to survive [10]. NIC has also been shown to affect various signal transduction pathways, such as Wnt/β-catenin, mammalian target of rapamycin (mTOR), STAT3, NFκB and Notch pathways [11]. Recent studies have also explored the potential of NIC as a therapeutic agent against cancer, bacterial or viral infections, and metabolic diseases. Studies have shown that NIC promotes autophagy in both small-cell lung carcinoma cells and mouse model, by inducing tumor cell death through the activation of autophagy and apoptosis via the AMPK/AKT/mTOR pathway [12]. NIC also inhibited viral propagation in a SARS-CoV-2 infected cell model by restoring autophagy, which had been reduced by virus infection [13]. Furthermore, NIC improves insulin and glucose homeostasis by activating autophagy in metabolic disease cells and mouse models [14].

Despite these promising results, no study has focused on its effects on aging. Therefore, in this study, we aimed to evaluate the effects of NIC on natural aging models. we report that NIC promotes healthy aging in *C. elegans* and mice. NIC increases physical function and mitochondrial function in skeletal muscles, which are reduced with aging. We found that NIC inhibited the expression of muscle atrophy-related genes by suppressing hyperactivated mTORC1 and enhancing autophagic flux, thereby improving age-related decline. Our results demonstrate a new function of the NIC in contributing to healthy aging, particularly skeletal muscle health.

## Materials and Methods

### *C. Elegans* strains and culture conditions

The *C. elegans* strains Bristol N2, TJ1060, VC2149, VC1878, CB1370, DA2121, BC12921, CB5600, and OP50 *Escherichia coli* (*E. coli*) were provided by the Caenorhabditis Genetics Center (University of Minnesota, MN, USA). The worms were maintained at 20 °C on nematode growth medium (NGM) agar plates seeded with a lawn of OP50 *E. coli*.

### Compound screening

We used the TJ1060 strain, which loses reproductive capacity when cultured above 25 °C to screen compounds and identify autophagy regulators [15]. NGM, an autophagy library compound (#BML-2837, Enzo, NY, USA), and concentrated OP50 *E. coli* were added to a 96 well plate which was dried to prepare a screening plate. Approximately 20 synchronized L1 worms were placed per well and incubated at 25 °C to induce aging. The M9 buffer was dispensed on day 10 of adulthood, the time point at which motor performance declines with age. Swimming performance of the worms was recorded to screen for compounds that could mitigate age-related decline in swimming performance.

### Lifespan analysis

Lifespan analysis was performed on synchronized eggs. The hatched eggs were grown in a normal NGM plate until young adults (about 12 h from larva stage 4). The young adult worms were transferred to NIC treated NGM (0, 25, 50, or 100 μM; crystallization of the compound occurred at concentrations above 200 µM, and no further increase in lifespan was observed) containing plates. The NGM was changed every 2 days and live, dead, or lost worms were counted.

### Pharyngeal pumping analysis

The number of contractions in the pharynx of the worms was counted for 10 s using an Olympus SZX7 zoom stereomicroscope (Olympus Corporation, Tokyo, Japan).

### Body bending analysis

The worms were placed in M9 buffer and bending was measured for 10 s.

### Motility analysis

Worms were treated with NIC, as in the lifespan analysis described above, and locomotion was measured on days 8, 10, 12, 14, 16, and 18 of adulthood. The locomotion phenotypes of the worms were determined by a mechanical stimulus with a picker and were used to classify the motility grades. Class A worms moved smoothly without touching and responded to prodding through vigorous movements. Class B worms did not move forward or backward even when prodded, but exhibited head and/or tail movement [16].

### Mitochondrial morphology analysis

Mitochondrial morphology was assessed using the CB5600 strain of *C. elegans*, which expresses GFP in the nucleus and mitochondria of the body wall muscle. Fluorescence was detected using an Olympus IX71 research inverted system microscope (Olympus Corporation; excitation wavelength: 460 ∼ 495 nm, emission wavelength: 510 nm). The mitochondrial network score was classified according to a previous study [17] in a blinded manner.

### Measurement of oxygen consumption rate (OCR) in *C. Elegans*

The OCR of *C. elegans* was measured using a Seahorse XF96 Analyzer (Agilent technologies, CA, USA) according to a previous study [18]. Fifteen synchronized worms (Day 2 or 8) were placed into XF96 wells. The baseline OCR was measured following which 100 μM FCCP was injected to induce maximal oxygen consumption. Subsequently, 40 mM sodium azide was injected to completely destroy the mitochondrial complex at the end of the experiment.

### Detection of intracellular reactive oxygen species (ROS)

Synchronized worms (Day 2 and 8) were transferred to M9 buffer containing 25 µM dichloro fluorescein diacetate (#35845, Sigma-Aldrich, MO, USA). After 30 min, the worms were imaged using an Olympus IX71 research inverted system microscope (Olympus Corporation; excitation wavelength: 460 ∼ 495 nm, emission wavelength: 510 nm).

### Age-pigment analysis

Worms (Day 2 and 8) were immobilized during imaging. Auto-fluorescence of the age pigment was assessed using an Olympus IX71 research inverted system microscope (Olympus Corporation; excitation wavelength: 340 ∼ 370 nm, emission wavelength: 460 nm).

### Mice experiments

C57BL/6N male mice were housed in a cage under constant conditions (12 h light/dark cycle; temperature, 22 ± 2°C; humidity, 50 ± 10 %). Two sets of mice experiments were conducted. The first set of mice was used to evaluate the effects of NIC on aging. Mice were raised until they were 12-month-old and divided into three groups (Old: normal AIN-93 M diet, Old + NL: 0.025 % NIC containing AIN-93 M diet, Old + NH: 0.050 % NIC containing AIN-93 M diet, n = 10). The intervention lasted nine months until mice were 21 months old, and several behavioral experiments were conducted before sacrifice. Six-month-old mice were considered as positive controls (Young). A second set of experiments was performed as additional experiments to determine the effects of NIC on the autophagic flux. Fourteen-month-old mice were fed a normal AIN-93 M diet or an AIN-93 M diet with 0.05 % NIC for 3 months (n = 6). Mice in each group (n = 3) were intraperitoneally injected with 30 mg/kg leupeptin (#L2884, Sigma-Aldrich) 4 h before sacrifice to examine autophagic flux in vivo.

### Frailty index measurement

The frailty index of the mice was measured as described by Whitehead, et al (2014) [19], by measuring 31 health-related variables. Tumor presence and size were accurately reevaluated during dissection, and body temperature and weight were measured as the mean and standard deviation of all mice.

### Treadmill test

Measurements were performed using a rodent treadmill (Ugo Basile, Gemonio, Italy). The mice ran at 10 m/min for the first 15 min at an incline of 5°. Subsequently, the speed increased to 2 m/min every 3 min. The mice were shocked if they left the lane, and the endpoint was set at 10 electric shocks.

### Grip strength test

Grip strength was measured three times using a GT3 grip strength test machine (Bioseb, Vitrolles, France), according to the manufacturer’s instructions.

### Y-maze test

The mice were positioned on one side of a Y-shaped acrylic container and video-recorded for 8 min. We tracked the movement of the mouse using the video and counted the movement only when the mice travelled more than half the distance of the arms.

### Protein isolation

Mice tissue and *C. elegans* were harvested using radio-immuno-precipitation assay buffer (#89900, Thermo Fisher Scientific, MA, USA) supplemented with inhibitor cocktails (#87786, Thermo Fisher Scientific). Samples were crushed using a Lysing Matrix D (#6540–434, MP Biomedicals, CA, USA) and a Fast Prep-24 System (MP Biomedicals). The supernatants were harvested after centrifuging at 13000 rpm for 10 min at 4 °C. The protein concentration in the lysate was calculated using a BCA assay kit (#23227, Thermo Fisher Scientific) and a Synergy H1 microplate reader (Agilent Technologies, USA).

### Western blot analysis

Isolated protein solutions of the same concentration and 5x dye SDS-PAGE loading buffer (#s2002; Biosesang, Gyeonggi-do, Republic of Korea) were mixed and heated at 95 °C for 5 min. Proteins were separated via SDS-PAGE using the PowerPac™ Supply (Bio-Rad, CA, USA) and subsequently transferred onto PVDF membranes (#162–0177, Bio-Rad) with the Turbo Transfer System (Bio-Rad). The membranes were blocked for 1 h in 5 % skimmed milk (Seoul milk corporation, Seoul, Republic of Korea) and prepared in Tris-buffered saline containing 0.1 % Tween 20 to minimize non-specific binding. They were then incubated overnight at 4 °C with primary antibodies. On the following day, the membranes were treated with secondary antibodies diluted in 5 % skimmed milk for 1 h at room temperature. Protein detection was performed using G:BOX Chemi XX6 imaging system (Syngene, Cambridge, UK) and Pierce ECL Substrate (#32106, Thermo Scientific). Band density was determined using the ImageJ software (National Institutes of Health). The list of used antibodies is shown in Supplementary Table 1.

### Transmission electron microscopy (TEM) imaging

Mouse skeletal muscle (gastrocnemius or soleus) was fixed with 2 % glutaraldehyde and 2 % paraformaldehyde in 0.1 M PBS. After dehydration and infiltration, the muscles were embedded. The samples were sectioned and collected on 100 mesh copper grids. After staining with 2 % uranyl acetate and lead citrate, the images were obtained using cryo-TEM (JEM-1400 Plus 120 kV, JEOL, Tokyo, Japan) at the Korea Basic Science Institute (Ochang, Republic of Korea).

### ATP content measurement

ATP content was measured using an ATPlite 1step Luminescence Assay System (#6016943, PerkinElmer, CT, USA). Briefly, the luminescence of samples with equal protein concentration was measured using a Synergy H1 microplate reader (Agilent Technologies) after reaction with reagents.

### Electron flow assay in isolated mitochondria

An electron flow assay was conducted as described previously [20]. Briefly, mitochondria isolated from the mouse gastrocnemius were seeded on to a XF96 plate with substrates (10 mM pyruvate, 2 mM malic acid, and 4 μM FCCP). The muscles were sequentially treated with 2 μM rotenone, 10 mM succinate, 4 μM antimycin A, and 100 mM ascorbate with 0.2 mM TMPD while measuring the OCR to investigate the complex-specific effects of NIC.

### Quantitative reverse transcription poly chain reaction (RT-qPCR)

RNA was extracted from mouse skeletal muscle or whole *C. elegans* using QIAzol reagent (#79306, Qiagen, Hilden, Germany) and an RNeasy mini kit (#74106, Qiagen). Complementary DNA was obtained using a ReverTra Ace™ master mix (#FSQ-201, Toyobo, Osaka, Japan) and thermal cycler (Bio-Rad) under the conditions: 37 °C for 15 min, 50 °C for 5 min, and then 98 °C for 5 min. A ViiA 7 real-time PCR system (Thermo Fisher Scientific) was used to analyze RT-qPCR with SYBR® Green master mix (#QPK-201, Toyobo). The primers used are listed in Supplementary Tables 2 and 3.

### Proteasome activity measurement

Proteasome activity was measured using the protocol described by Kaiser, et al (2022) [21]. Briefly, 3-Substrate system Proteasome-GloTM Assay (#G8531, Promega, WI, USA) was used to measure the activity of each proteasome in the lysate of gastrocnemius muscles. MG-132 50  µM (#474790, Sigma-Aldrich), which is a proteasome inhibitor, was used to background.

### mRNA-seq analysis

Libraries were generated from total RNA using the CORALL RNA-Seq V2 library preparation kit (LEXOGEN, Vienna, Austria). mRNA was extracted utilizing the poly(A) RNA selection kit (LEXOGEN), and the isolated mRNAs were subsequently used for cDNA synthesis and fragmentation, following the manufacturer’s protocol. Indexing was conducted with Illumina indexes 1–12, and PCR amplification was performed to enrich the libraries. The quality and fragment size of the libraries were assessed using the TapeStation HS D1000 Screen Tape (Agilent Technologies). Quantification was carried out using a library quantification kit in conjunction with the StepOne Real-Time PCR System (Thermo Fisher Scientific). High-throughput sequencing was performed using paired-end 100 sequencing on the NovaSeq 6000 platform (Illumina, CA, USA). Quality control of the raw sequencing data was performed using FastQC, while adapter sequences and low-quality reads were removed with Fastp. The processed reads were aligned to the reference genome using STAR, and quantification of gene expression levels was conducted with Salmon. Read count normalization was performed using the TMM + CPM method implemented in the Python package “conorm.” Data analysis and graphical visualization were conducted with ExDEGA (Ebiogen, Seoul, Korea).

### Statistical analysis

All statistical analyses were performed using the GraphPad Prism 10 software. Differences between groups were assessed using one-way or two-way ANOVA. Data are presented as mean ± standard error of the mean. Significance levels of *p < 0.05, **p < 0.01, and ***p < 0.001 were considered to indicate differences between the groups.

### Ethics statement

All experiments involving animals were conducted according to the ethical policies and procedures approved by the Animal Care and Use Committee of Korea Food Research Institute (Approval no. KFRI-M−22064).

## Results

### Niclosamide extends lifespan with health span in *C. Elegans*

**Fig. 1:**
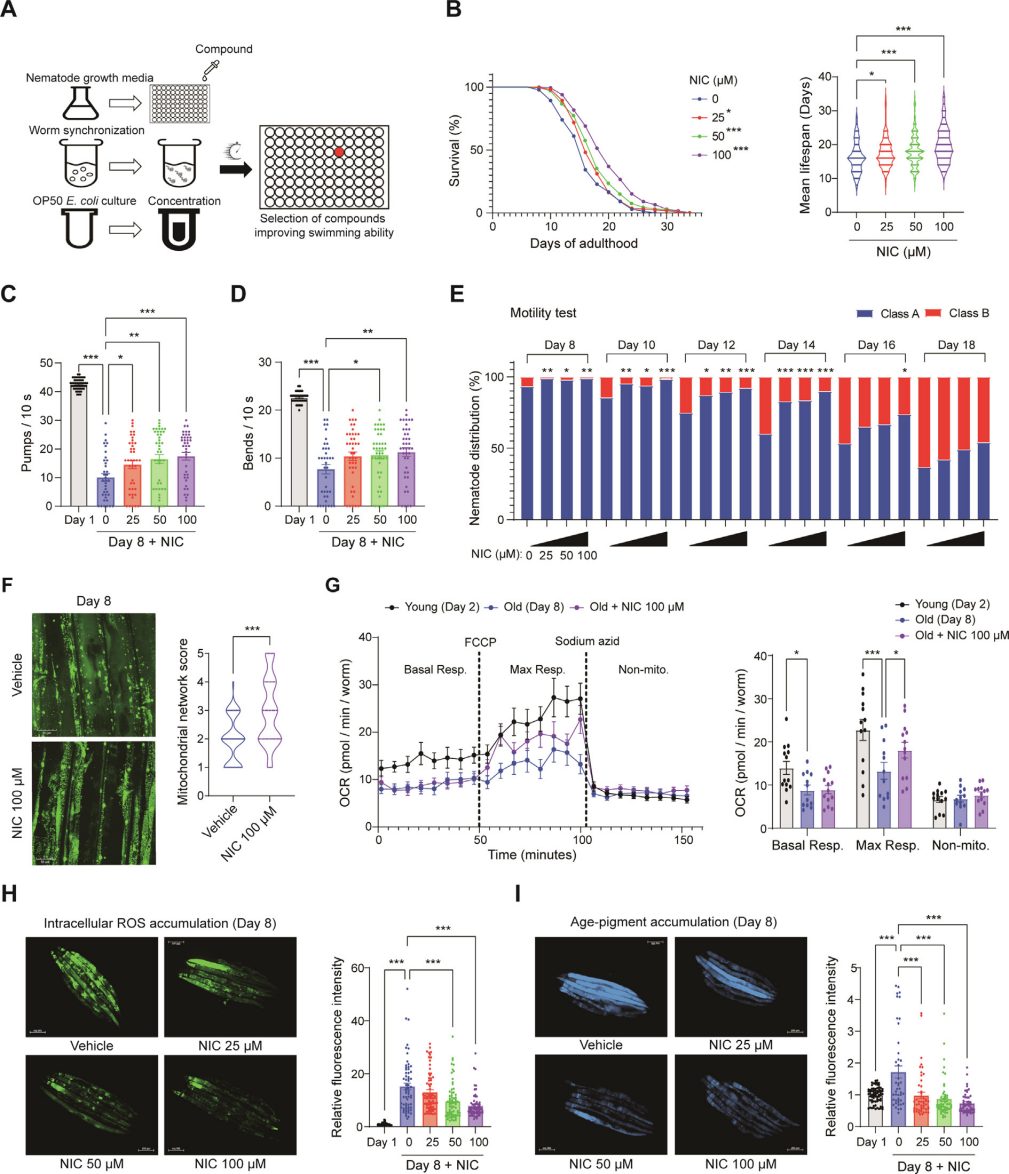
**Niclosamide extends lifespan with health span in *C. elegans.*** Wild-type N2 worms were treated with 0, 25, 50, or 100 μM NIC. (**A**) Experimental design for compound screening. (**B**) Effect of NIC on lifespan and mean lifespan. The effect of NIC on (**C**) Pharyngeal pumping, (**D**) Body bending, and (**E**) Motility. (**F**) CB5600 strain worms were treated with 0 or 100 μM NIC and mitochondrial morphology was evaluated (Scale bar: 50 µm). (**G**) The Effect of NIC on OCR. Effects of NIC on (**H**) Intracellular ROS accumulation and (**I**) Age-pigment accumulation (scale bar: 200 µm). ANOVA and Kaplan–Meier survival analyses were used for statistical analysis. NIC: Niclosamide, OCR: Oxygen consumption rate, Resp: Respiration, Non-mito: Non-mitochondrial respiration, ROS: Reactive oxygen species, * p < 0.05, ** p < 0.01, *** p < 0.001.

We used the TJ1060 strain of *C. elegans* to screen compounds and identify autophagy regulators that improve age-related decline in swimming ability. A total 97 compounds were tested at a concentration of 50 µM, and several compounds, including NIC, valproic acid, LY294002, thapsigargin, calcimycin, plumbagin, and rockout were found to improve the reduced swimming performance (Fig. 1A, Supplementary video). Among these compounds, NIC was selected because it had the highest efficacy, novelty, and accessibility. Therefore, its effects on lifespan and health span were further evaluated. The wild-type N2 strains were treated with different NIC concentrations (0, 25, 50, or 100 µM) to analyze their effects on lifespan. NIC treatment increased the lifespan in a dose-dependent manner, with the mean lifespan extended by 8.11 %, 12.19 % and 21.67 % for 25, 50 and 100 µM NIC treated worms, respectively (Fig. 1B). Subsequently, we measured health span biomarkers, such as pharyngeal pumping, body bending, motility, mitochondrial morphology and function, age–pigment accumulation, and intracellular ROS levels, to evaluate the effects of NIC on *C. elegans* health span. Pharyngeal pumping increased with NIC treatment in a dose-dependent manner. The mean pharyngeal pumping in the vehicle group was 10.05, whereas treatment with 25, 50 and 100 µM NIC increased the mean pharyngeal pumping to 14.56, 16.53 and 17.49, respectively (Fig. 1C). NIC treatment also increased body bending in a dose-dependent manner, with the mean body bending increasing from 7.70 in the vehicle to 10.39, 10.67, and 11.27 with 25, 50, and 100 µM NIC, respectively (Fig. 1D).

Motility analysis showed that NIC treatment increased the ratio of class A worms (healthy state) on the final (Day 18) from 36.67 % in the vehicle to 42.00 %, 49.12 %, and 54.12 % in the 25, 50, and 100 µM NIC treated groups, respectively (Fig. 1E). Additionally, 100 µM NIC treatment improved the mitochondria network score to 2.96 compared to 1.96 in the vehicle, indicating that relatively less mitochondria damage occurred (Fig. 1F). Moreover, OCR measurements showed that treatment with 100 µM NIC significantly increased maximal respiratory capacity compared to the vehicle, indicating that NIC improved mitochondrial function impaired by aging (Fig. 1G). NIC treatment also reduced intracellular ROS accumulation in a dose-dependent manner, with a reduction of 13.76 %, 36.57 %, and 48.22 % in the 25, 50, and 100 µM NIC-treated groups, respectively, compared to the vehicle (Fig. 1H). Furthermore, NIC treatment decreased age-pigment accumulation by 46.46 %, 51.99 %, and 57.95 % in the 25, 50, and 100 µM NIC-treated groups, respectively, compared to the vehicle (Fig. 1I). Collectively, these results suggest that NIC treatment increases the lifespan and the health span in *C. elegans*.

### Niclosamide improves frailty phenotypes associated with aging in mice

**Fig. 2:**
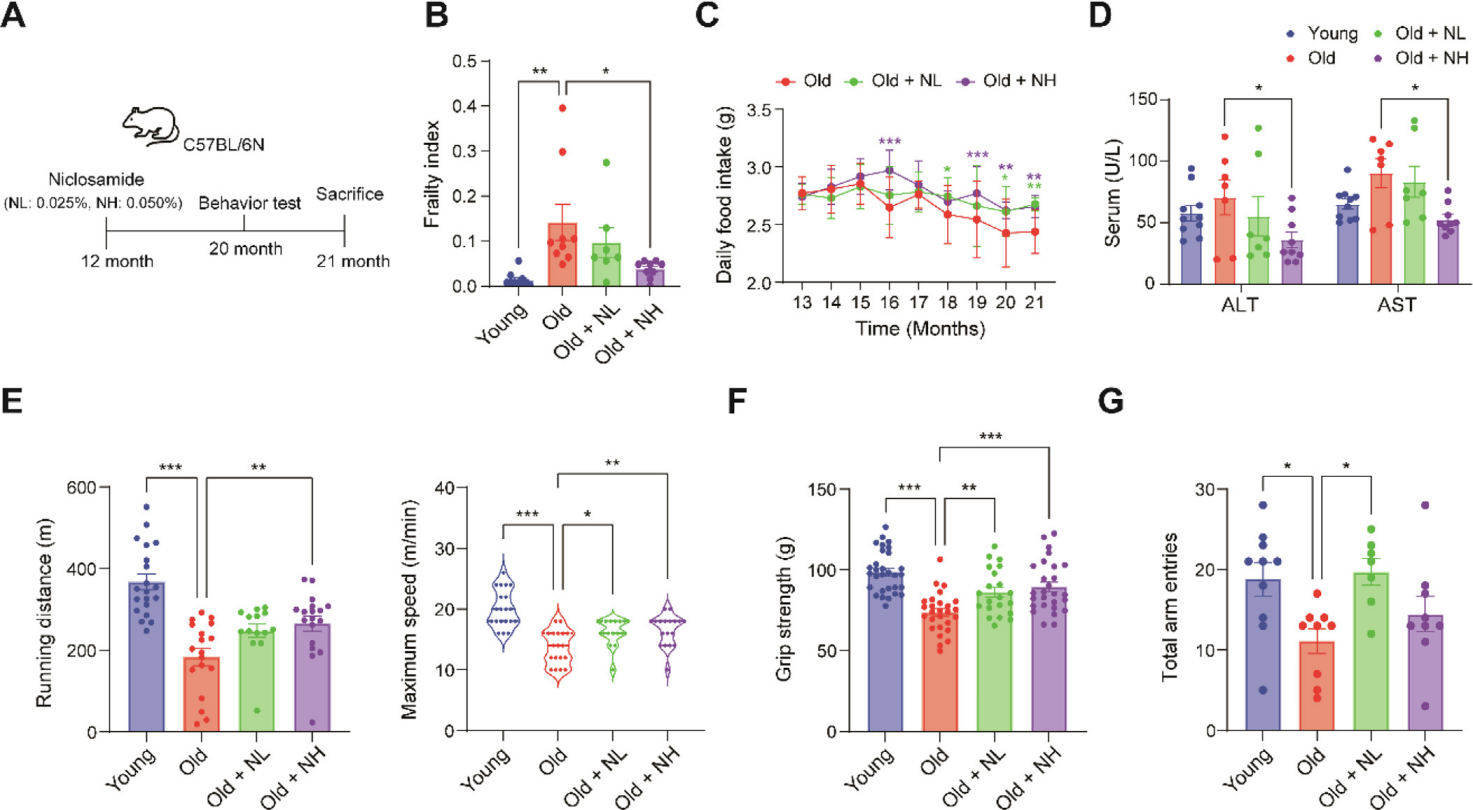
**Niclosamide improves frailty phenotypes associated with aging in mice.** NIC (0 %, 0.025 %, or 0.05 %) was included in the diet and administered for 9 months from 12 – 21 months of age. Six-month-old mice were used as the young group. (**A**) Experimental design. Effect of NIC on (**B**) Frailty index, (**C**) Daily food intake, and (**D**) Serum alanine aminotransferase (ALT) and aspartate transaminase (AST) levels. (**E**) Effect of NIC on Running distance and Maximum speed in the treadmill test. Effects of NIC on (**F**) Grip strength and (**G**) Total arm entries in the Y-maze test. One-way or two-way ANOVA used for statistical analysis. NIC: Niclosamide, NL: Niclosamide low dose treated group (0.025 %), NH: Niclosamide high dose treated group (0.05 %), * p < 0.05, ** p < 0.01, *** p < 0.001.

Subsequently, we evaluated whether the effects of NIC observed in *C. elegans* were also observed in a mammalian model. Mice were administered NIC mixed with the AIN-93 M diet at low (0.025 %, NL) and high (0.050 %, NH) doses. The intervention started at 12 months and continued until 21 months (Fig. 2A). During the intervention, three mice in the old group, three in the NL group, and one in the NH group died (Supplementary Fig. 1). The frailty index significantly increased from 0.014 in the young group to 0.142 in the old group but decreased to 0.097 in the NL group and significantly decreased to 0.038 in the NH group (Fig. 2B). Food intake began to decline rapidly at 18 months of age in the old group. However, NIC treatment significantly mitigated this decline (Fig. 2C). Despite the difference in food intake, both the mean body weight and fat mass ratio were lower in the NIC treatment groups than the old group (Supplementary Fig. 2A-C). Histological analysis of the liver and EP fat revealed that the number and size of lipid droplets were significantly lower in both NIC treatment groups than the old group (Supplementary Fig. 2D, E). Correspondingly, mRNA expression levels of Srebp1c, a transcription factor associated with age-related lipid accumulation [22,23], along with its target genes Acc and Fasn, were significantly elevated only in the liver and EP fat of the old group (Supplementary Fig. 4A, B). These results suggest that NIC is involved in lipid metabolism and mitigates age-related to fat accumulation.

We also observed a significant decrease in the hepatic protein levels of P16, P21, and P53, which were known senescent cell markers associated with the induction of hepatic steatosis [24], specifically in the NH group (Supplementary Fig. 5A). Furthermore, using Oil Red O staining and triglyceride quantification, we observed that fat invasion in the skeletal muscles was reduced in the NIC treatment groups compared to the old group, similar to the findings in the liver (Supplementary Fig. 2F, G). Moreover, the serum levels of alanine aminotransferase (ALT) and aspartate aminotransferase (AST), indicators of hepatotoxicity, were significantly reduced in the NH group compared to the old group, without increasing after 9 months of prolonged NIC administration (Fig. 2D).

Several behavioral tests were conducted to evaluate physical function. The average running distance on the treadmill decreased from 367.57 m in the young group to 183.59 m in the old group, but improved to 248.07 m in the NL group and 265.93 m in the NH group. Maximum speed decreased from 20.10 m/min in the young group to 13.67 m/min in the old group, but significantly improved to 16.14 m/min in the NL group and 16.56 m/min in the NH group (Fig. 2E). Grip strength decreased from 98.76 g in the young group to 73.61 g in the old group, but significantly improved to 86.04 g in the NL group and 89.43 g in the NH group (Fig. 2F). Total arm entries, as measured by the Y-maze test, decreased from 18.80 in the young group to 11.11 in the old group, indicating reduced physical activity with aging. This decline was reversed in the NIC-treated groups, with entries increasing to 19.71 in the NL group and 14.44 in the NH group (Fig. 2G, Supplementary Fig. 3). Collectively, these results suggest that NIC attenuates frailty phenotypes.

### Niclosamide improves skeletal muscle aging

**Fig. 3:**
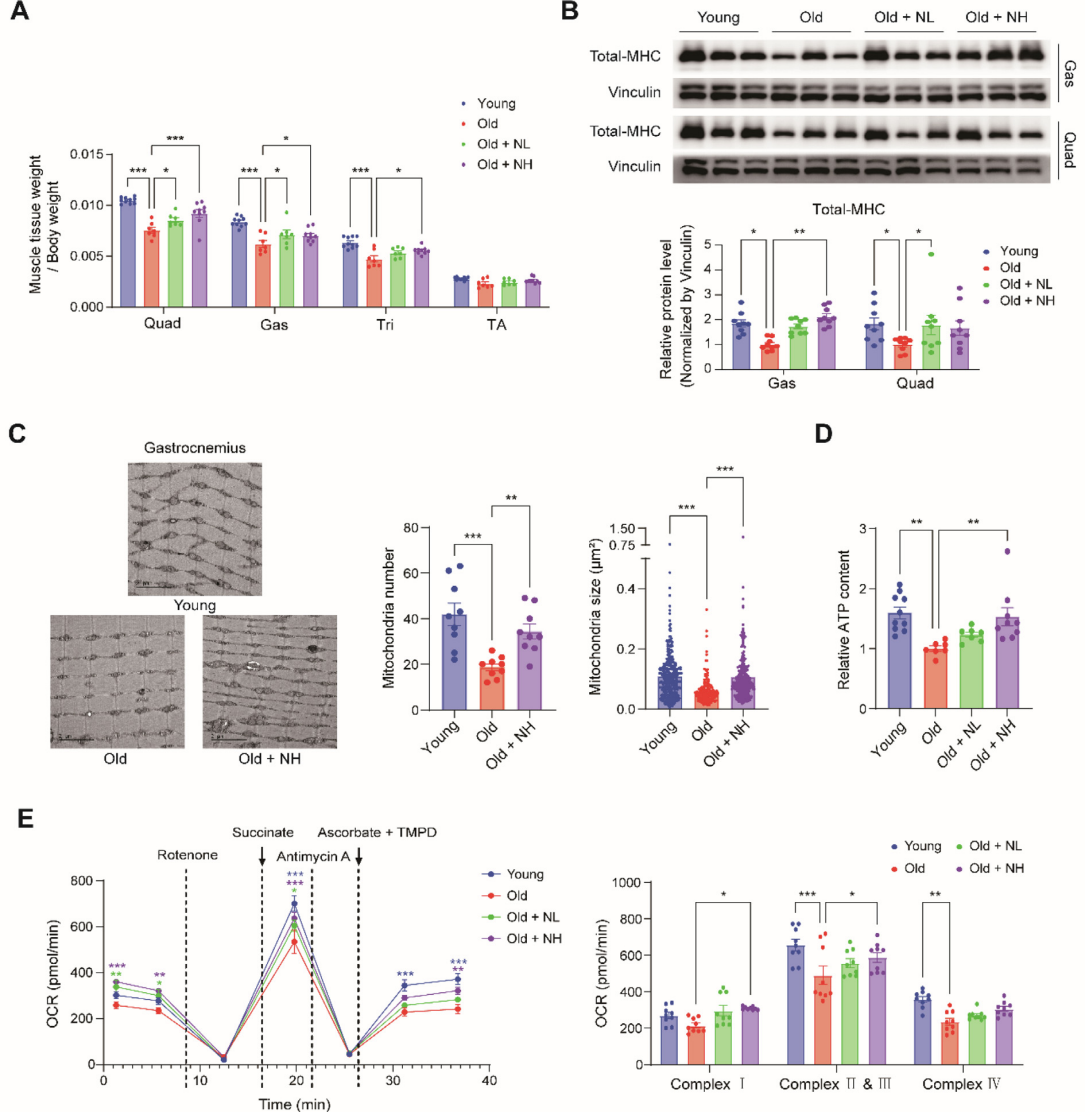
**Niclosamide improves skeletal muscle aging.** NIC (0 %, 0.025 %, or 0.05 %) was included in the diet and administered for 9 months from 12 – 21 months of age. Six-month-old mice were used as the young group. (**A**) Muscle tissue weight was normalized to the body weight. (**B**) Western blot analysis of total myosin heavy chain (MHC) in the gastrocnemius and quadriceps muscle tissues. Effect of NIC on (**C**) Mitochondrial morphology assessed via TEM imaging (Scale bar: 2 µm) and (**D**) ATP content in Gas. (**E**) Effect of NIC on OCR in Gas-isolated mitochondria. One-way or two-way ANOVA used for statistical analysis. NIC: Niclosamide, Quad: Quadriceps muscle, Gas: Gastrocnemius muscle, Tri: Triceps brachii muscle, TA: Tibialis anterior muscle, NL: Niclosamide low dose treated group (0.025 %), NH: Niclosamide high dose treated group (0.05 %), OCR: Oxygen consumption rate, * p < 0.05, ** p < 0.01, *** p < 0.001.

Given our observation that NIC mitigates the reduction in physical function associated with aging, we evaluated the effect of NIC on the skeletal muscles. We found that the relative mass of skeletal muscle tissues was reduced in the old group compared to the young group, and this reduction was significantly reversed in the NIC treatment groups in the quadriceps, gastrocnemius, and triceps (Fig. 3A). Furthermore, NIC treatment reversed the decrease of total myosin heavy chain (MHC) levels in the quadriceps and gastrocnemius muscles of aged mice (Fig. 3B). Additionally, we observed that the protein levels of P16, P21, and P53, markers of senescent cell accumulation, were significantly reduced in NIC-treated groups compared to the old group (Supplementary Fig. 5B). These findings suggested that NIC treatment plays a beneficial role in maintaining skeletal muscle mass during aging.

Next, we evaluated the morphology and function of the mitochondria, which play a major role in skeletal muscle function. The number of mitochondria in the same area significantly decreased from 41.89 in the young group to 18.67 in the old group but improved to 34.33 in the NH group. The average size of mitochondria was reduced from 0.11 μm^2^ in the young group to 0.06 μm^2^ in the old group, but increased to 0.11 μm^2^ in the NH group (Fig. 3C). To further evaluate mitochondrial function, we measured the ATP content in the tissue and OCR in isolated mitochondria. The relative ATP content was 1.60-fold in the young group, 1.23-fold in the NL group, and 1.53-fold in the NH group compared to the old group (Fig. 3D). OCR measurements showed that complex 1 activity decreased from 269.52 pmol/min in the young group to 214.99 pmol/min in the old group and improved to 295.57 pmol/min in the NL group and 308.70 pmol/min in the NH group. Complex II & III activity significantly decreased from 656.00 pmol/min in the young group to 490.59 pmol/min in the old group and improved to 556.30 pmol/min in the NL group and 587.53 pmol/min in the NH group. Complex IV activity significantly decreased from 357.85 pmol/min in the young group to 235.03 pmol/min in the old group and improved to 270.03 pmol/min in the NL group and 305.68 pmol/min in the NH group (Fig. 3E). Although NIC has been identified in previous studies as a mitochondrial uncoupler [25,26], we detected no alterations in OXPHOS complex protein levels and in the mRNA expression of *Ucp2* and *Ucp3* by NIC treatment, indicating that NIC did not act as a mitochondrial uncoupler at the applied dose (Supplementary Fig. 6, 7). Collectively, these results suggest that NIC ameliorates skeletal muscle deterioration associated with aging.

### Niclosamide improves muscle atrophy-related ubiquitin–proteasome system induced by mTORC1 hyperactivity

**Fig. 4:**
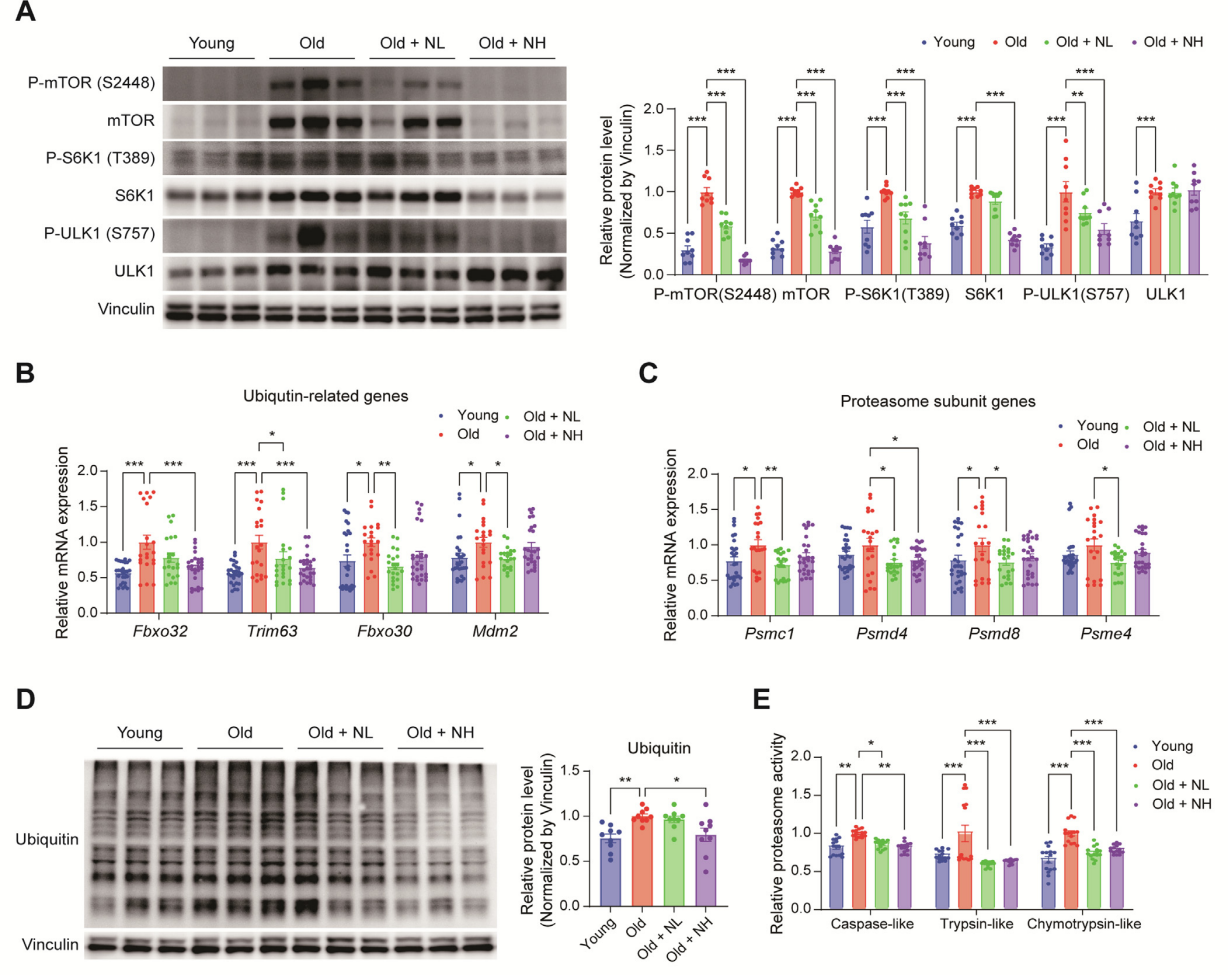
**Niclosamide improves muscle atrophy-related ubiquitin**–**proteasome system induced by mTORC1 hyperactivity.** NIC (0 %, 0.025 %, or 0.05 %) was added to the diet and administered for 9 months from 12 – 21 months of age. Six-month-old mice were used as the young group. The gastrocnemius muscle was used for the analysis. (**A**) Western blot analysis of P-mTOR (S2448), mTOR, P-S6K1 (T389), S6K1, P-ULK1 (S757), and ULK1. Effect of NIC on the mRNA levels of (**B**) Ubiquitin-related genes (*Fbxo32, Trim63, Fbxo30,* and *Mdm2*) and (**C**) Proteasome subunit genes (*Psmc1, Psmd4, Psmd8*, and *Psme4*) (**D**) Western blotting analysis of ubiquitin levels. (**E**) The Effect of NIC on proteasome activity. One-way or two-way ANOVA used for statistical analysis. NIC: Niclosamide, NL: Niclosamide low dose treated group (0.025 %), NH: Niclosamide high dose treated group (0.05 %), * p < 0.05, ** p < 0.01, *** p < 0.001.

We investigated the mechanisms underlying the effects of NIC. P-mTOR (S2448), P-S6K1 (T389), and P-ULK1 (S757) protein levels, which indicate mTORC1 activity, were measured, based on the results of a previous study which showed that NIC inhibits mTOR [27]. Elevated mTOR expression was discernible in the old group compared to the young group, which increased the phosphorylation of its subsequent targets, P-S6K1 (T389) and P-ULK1 (S757). P-mTOR (S2448), P-S6K1 (T389), and P-ULK1 (S757) protein levels were 0.30-fold, 0.58-fold, and 0.34-fold lower, respectively, in the young group than in the old group. Moreover, P-mTOR (S2448), P-S6K1 (T389), and P-ULK1 (S757) levels were reduced by 0.59-fold, 0.68-fold, and 0.75-fold in the NL group, and 0.17-fold, 0.39-fold, and 0.55-fold in the NH group, respectively (Fig. 4A). These results suggest that NIC inhibits hyperactivated mTORC1 due to aging in skeletal muscle.

Next, we measured the mRNA expression levels of muscle proteostasis-related genes that are regulated by the mTORC1 pathway [21]. The mRNA expression levels of *Fbxo32*, *Trim63*, *Fbxo30*, and *Mdm2*, which are ubiquitin-related genes regulated by mTORC1, were significantly increased in the old group, and these increases were ameliorated by NIC treatment (Fig. 4B). The expression of *Psmc1*, *Psmd4*, *Psmd8*, and *Psme4*, which are proteasome subunit genes regulated by mTORC1, was also increased in the old group and was decreased by NIC treatment (Fig. 4C). Furthermore, immunoblotting using anti-ubiquitin antibodies showed that the muscles of the old group contained more ubiquitinated proteins than those of the young group (Fig. 4D). Several studies have shown that an increase in ubiquitinated proteins in aged muscles contributes to the deterioration of protein homeostasis [21,28]. Therefore, we examined proteasome activity and found it to be higher in the old group than that in the young group. However, the increased activity was reduced by NIC treatment (Fig. 4E). Collectively, these results suggest that NIC effectively suppresses accelerated muscle atrophy during aging by regulating the expression of ubiquitin and proteasome genes induced by mTORC1 hyperactivation.

### Niclosamide improves autophagic flux in aged muscle

**Fig. 5:**
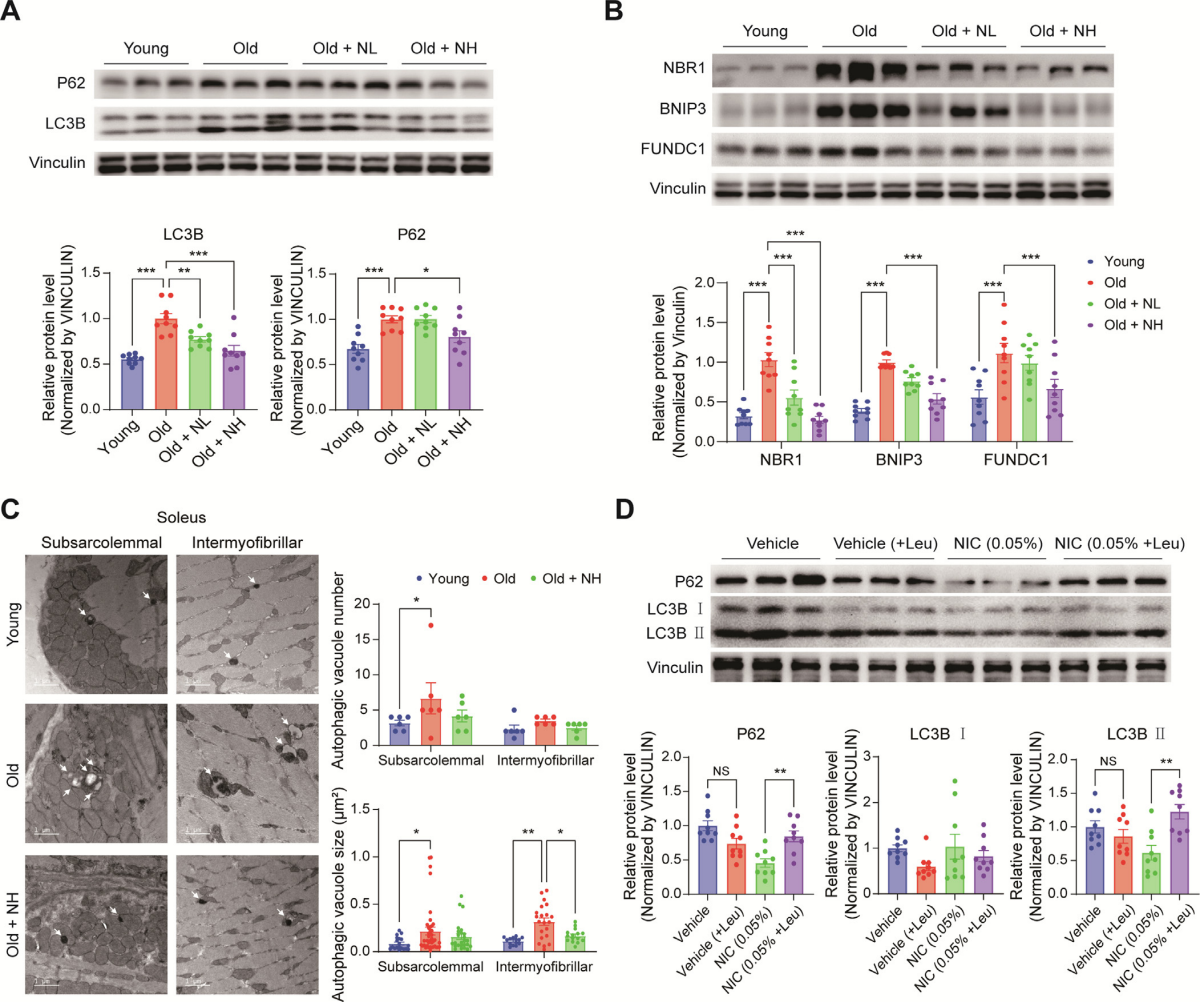
**Niclosamide improves autophagic flux in aged muscle.** NIC (0 %, 0.025 %, or 0.05 %) was added to the diet and administered for 9 months from 12 – 21 months of age. Six-month-old mice were used as the young group. Western blot analysis of (**A**) P62 and LC3B, and (**B**) NBR1, BNIP3, and FUNDC1 in the gastrocnemius muscles. (**C**) Effect of NIC on morphology of autophagic vacuoles through TEM imaging in soleus muscle (Scale bar: 1 µm). (**D**) NIC (0 % or 0.05 %) was added to the diet and administered for 3 months, from 14 – 17 months of age. Half of the mice in each group were intraperitoneally injected with 30 mg/kg leupeptin 4 h before sacrifice. Western blot analysis of P62 and LC3B in gastrocnemius muscles. One-way or two-way ANOVA used for statistical analysis. NIC: Niclosamide, NL: Niclosamide low dose treated group (0.025 %), NH: Niclosamide high dose treated group (0.05 %), +Leu: 30 mg/kg leupeptin intraperitoneal injection, NIC (0.05 %): Niclosamide high dose treated group (0.05 %), NS: Not significant, * p < 0.05, ** p < 0.01, *** p < 0.001.

Subsequently, we also analyzed the activity of autophagy, another proteostasis system inhibited by mTORC1. LC3B and P62 protein levels were measured to evaluate the activity of autophagy. LC3B and P62 protein levels were 0.55-fold and 0.67-fold lower, respectively, in the young group compared to the old group. In the NIC treatment groups, these levels were 0.77-fold and 1.00-fold in the NL group and 0.65-fold and 0.81-fold in the NH group (Fig. 5A), indicating that the expression of LC3 and P62 significantly increased with aging. Furthermore, we measured the protein levels of other autophagy receptors and cargo markers, including NBR1, BNIP3, and FUNDC1. Their protein levels increased with aging but were significantly reduced in the NIC treatment groups (Fig. 5B). Subsequently, we observed the morphology of the autophagic vacuoles using TEM imaging of the soleus. We observed significantly larger autophagic vacuoles in the old group than in the young group in both the subsarcolemmal and intermyofibrillar areas of the skeletal muscle, which were attenuated in the NH group (Fig. 5C, Supplementary Fig. 8). These results suggest that the age-related increase in LC3B levels may be due to an increase in the formation of large autophagic vacuoles.

Consequently, we performed additional experiments in which 14-month-old middle-aged mice were fed a 0.05 % NIC containing diet for 3 months and injected with leupeptin, which inhibits autophagic flux, 4 h before sacrifice to further investigate the effects of NIC on autophagy (Supplementary Fig. 9A). We analyzed P62 and LC3B protein expression to assess autophagic flux in the gastrocnemius of the additional experimental mouse set. Although the relative P62 protein level in the vehicle group did not significantly change from 1-fold to 0.74-fold upon leupeptin treatment, it significantly increased from 0.46-fold to 0.85-fold in the NIC treatment group. In addition, the level of the LC3B II form, which forms autophagosomes, did not significantly change from 1-fold to 0.86-fold upon leupeptin treatment in the vehicle group, but was significantly increased from 0.62-fold to 1.23-fold in the NIC treatment group (Fig. 5D). These results suggest that autophagic flux is impaired in aged skeletal muscle and that NIC treatment ameliorates it.

### Niclosamide ameliorates hyperactive LET-363/mTOR and improves autophagic flux in *C. Elegans*

**Fig. 6:**
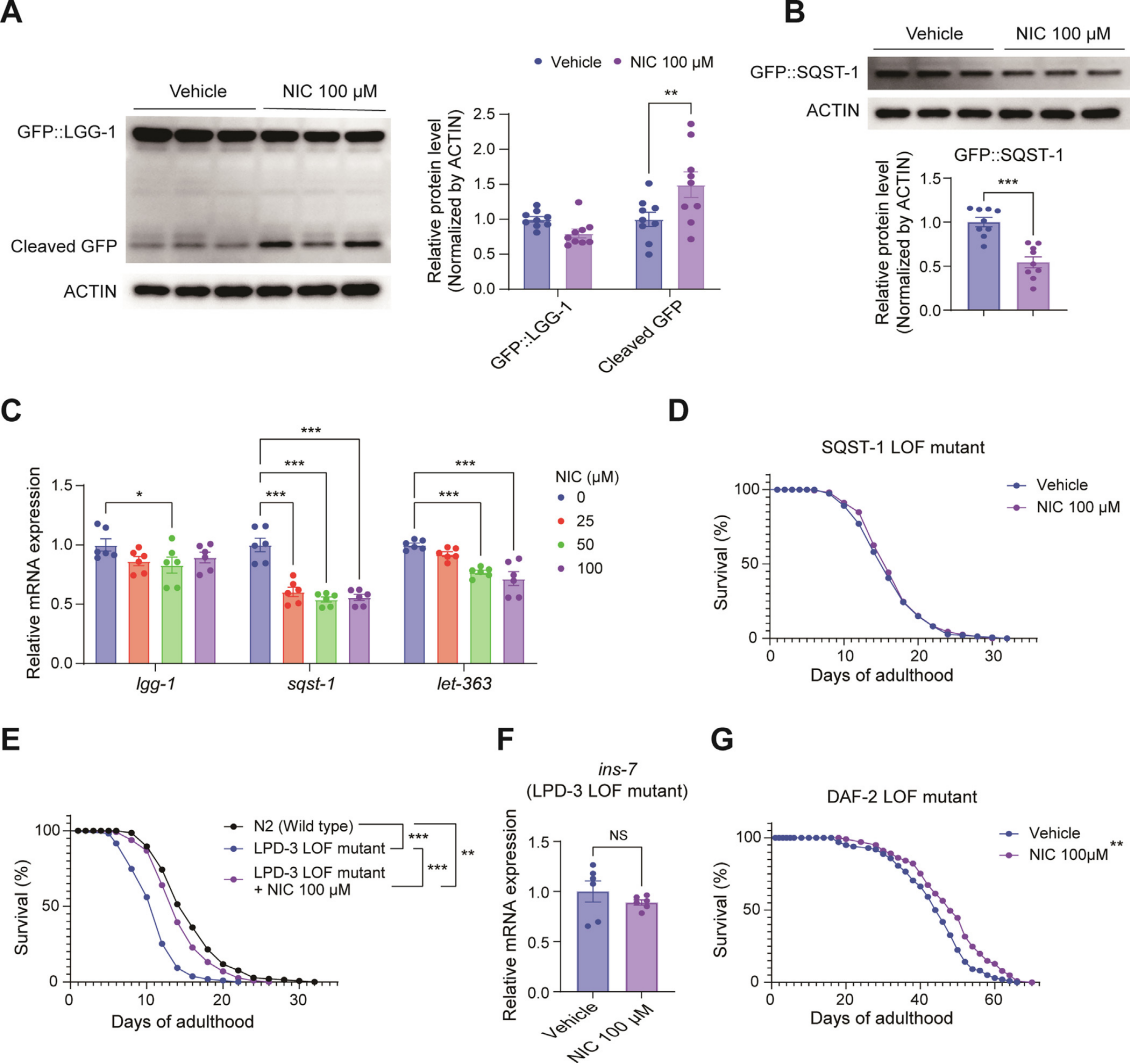
**Niclosamide ameliorates hyperactive LET-363/mTOR and improves autophagic flux in *C. elegans.*** (**A**) DA2121 strain worms were treated with 0 or 100 μM NIC for 8 days. Western blot analysis for LGG-1. (**B**) BC12921 strain worms were treated with 0 or 100 μM NIC for 8 days. Western blot analysis for SQST-1. (**C**) N2 wild-type worms were treated with 0, 25, 50, or 100 μM NIC for 8 days. Effect of NIC on mRNA level of *lgg-1, sqst-1,* and *let-363*. (**D**) VC2149 strain worms were treated with 0 or 100 μM NIC. Effect of NIC on lifespan in SQST-1 LOF mutant. (**E**) VC1878 strain worms were treated with 0 or 100 μM NIC. The wild-type N2 strain was used as a normal control. Effect of NIC on lifespan in LET-363 hyperactivity mutant. (**F**) VC1878 strain worms were treated with 0 or 100 μM NIC for 5 days. Effect of NIC on mRNA level of *ins-7*. (**G**) CB1370 strain worms were treated with 0 or 100 μM NIC. Effect of NIC on lifespan in DAF-2 LOF mutant. ANOVA and Kaplan-Meier survival analysis were used for statistical analysis. NIC: Niclosamide, LOF: Loss of function, NS: Not significant, * p < 0.05, ** p < 0.01, *** p < 0.001.

Thereafter, we determined whether NIC, a known autophagy inducer, promotes autophagy in *C. elegans*. We used transgenic worms expressing GFP::LGG-1/LC3 or GFP::SQST-1/P62 to determine the effects of NIC on autophagy. These worms were treated with NIC 0 or 100 µM. The results showed that NIC did not increase the expression of LGG-1/LC3, but increased the expression of cleaved GFP, which occurs as a result of autophagy, by 1.50-fold compared to the vehicle group (Fig. 6A). Furthermore, NIC treatment decreased the accumulation of SQST-1/P62, a receptor that labels the material to be degraded, by 0.54-fold compared to the vehicle group (Fig. 6B). In addition, we evaluated *lgg-1*/*Lc3*, *sqst-1*/*P62* and *let-363*/*mtor* mRNA expression levels in wild-type worms treated with NIC 0, 25, 50 or 100 µM. The *lgg-1*/*Lc3* mRNA expression level was not significantly different from that in the vehicle group, whereas *sqst-1*/*P62* mRNA expression was significantly reduced by 0.60-fold, 0.54-fold, and 0.56-fold, in the 25, 50 or 100 µM NIC treatment groups, respectively. Additionally, *let-363*/*mtor* mRNA expression levels were reduced in a dose-dependent manner to 0.92-fold, 0.77-fold, and 0.72-fold, in the 25, 50 or 100 µM NIC treatment groups, respectively, compared to the vehicle group (Fig. 6C). Moreover, NIC treatment did not increase the lifespan of SQST-1/P62 loss of function (LOF) mutant worms, indicating that SQST-1/P62, which is involved in autophagy, is essential for the lifespan-extending effect of NIC (Fig. 6D).

Next, we evaluated whether NIC could inhibit LET-363/mTOR in a model of LET-363/mTOR hyperactivity, thereby alleviating several exacerbations following the hyperactivation of LET-363/mTOR. We used a model of LET-363/mTOR hyperactivation upon overexpression of INS-7, which is an agonist of insulin, following the loss of LPD-3 function, proposed by Pandey, et al (2024) [29]. This model exhibits several negative phenotypes, including a short lifespan and an accelerated decline in physical function. The lifespan of the LPD-3 LOF mutant was reduced to 70.51 % compared to the wild-type, and NIC treatment significantly rescued it to 91.47 % (Fig. 6E). Furthermore, NIC treatment did not affect *ins-7* gene expression levels and significantly increased the lifespan of the DAF-2 LOF mutant (Fig. 6F, G). These results suggest that NIC does not exert its effects by inhibition of *ins-7* or upstream insulin/IGF-1 signaling, but rather by inhibiting the hyperactivation of LET-363/mTOR.

Collectively, these results demonstrate that NIC could ameliorate the lifespan shortening caused by LET-363/mTOR hyperactivation, and that it could ameliorate the age-related decline of autophagic flux in *C. elegans* as in mice.

### Gene expression profile in Niclosamide treated quadriceps muscles

**Fig. 7:**
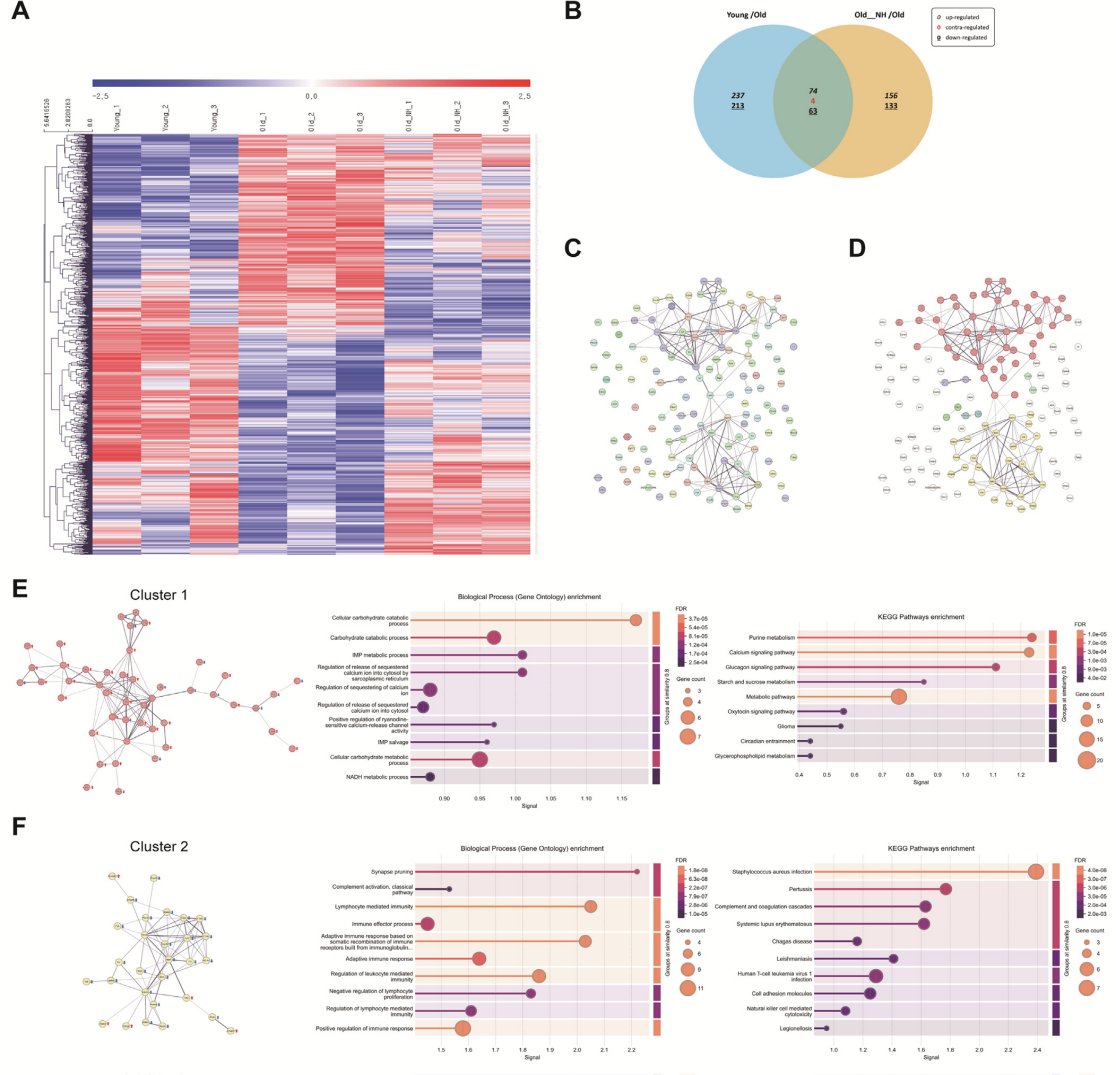
**Gene expression profile in niclosamide treated quadriceps muscles.** NIC (0 % or 0.05 %) was added to the diet and administered for 9 months from 12 – 21 months of age. Six-month-old mice were used as the young group. Gene expression profiles in quadriceps muscles were analyzed. (**A**) Heat map and (**B**) Venn diagram of genes with significant differences in expression compared to the old group. (**C**) Functional protein association network analysis, and (**D**) Clustering results obtained using STRING for common genes. Functional protein association networks and enrichment of biological processes and KEGG pathways of (**E**) Cluster 1 and (**F**) Cluster 2. NIC: Niclosamide, NH: Niclosamide high dose treated group (0.05 %).

We performed mRNA-seq analysis of the quadriceps and obtained gene expression profiles to further analyze the changes caused by NIC treatment during aging. A total of 880 genes were selected with a more than 1.3-fold difference and p ≤ 0.05 in the young or NH group than that in the old group. These genes were visualized using heat maps. Many of the genes with opposite expression levels between the young and old groups tended to be less different in the NH group or matched the expression pattern in the young group (Fig. 7A). Among the 880 genes, 141 genes overlapped in the young and NH groups, exhibiting consistent expression patterns, except for four genes (Fig. 7B). We analyzed the functional protein association networks using STRING and the 137 genes with common expression patterns in the young and NH groups. A network between proteins was identified, and clustering analysis was performed (Fig. 7C, D). Cluster analysis yielded two main clusters. The proteins in cluster 1 were mainly involved in metabolism or muscle contraction, and their expression was generally higher than the old group (Fig. 7E). The results of an additional set of mouse experiments support these analyzed results. In 14-month-old middle-aged mice, a 3-month NIC 0.05 % diet resulted in a faster normalization of blood glucose in the oral glucose tolerance test, compared to the vehicle group, although this was not significant (Supplementary Fig. 9D). Furthermore, energy expenditure, which was calculated from the changes in O_2_ and CO_2_, was significantly higher in the NIC treatment group than that in the vehicle group (Supplementary Fig. 9E-G).

The proteins in cluster 2 were mainly related to immunity and inflammation, and their expression was generally lower than the old group (Fig. 7F). Additionally, the levels of TNF-α and IL-6 in skeletal muscle were significantly reduced in the NH group compared to the old group (Supplementary Fig. 13). In addition to genes that overlapped with the young group, we analyzed genes that were differentially expressed in response to NIC treatment compared to the old group. We specifically examined the expression patterns of genes associated with the Wnt/β-catenin, mTORC1, STAT3, NF-κB, and Notch signaling pathways, which are known to be inhibited by NIC [11]. Although not all genes showed statistical significance, a substantial number exhibited expression patterns consistent with pathway inhibition in the NH groups (Supplementary Fig. 10). These pathways are closely linked to inflammation and cancer, and the tumor incidence observed at the time of sacrifice (two cases in the old group, one in the NL group, and none in the NH group) suggests that NIC may have the potential to suppress age-related inflammation and tumorigenesis.

Additionally, GO biological process analysis of differentially expressed genes in aged mice following NIC treatment revealed a significant downregulation of genes associated with cell migration and positive regulation of angiogenesis in the NH groups. KEGG pathway analysis further showed notable changes in the expression of genes involved in proteoglycans in cancer and pathways in cancer (Supplementary Fig. 11).

These results suggest that NIC mitigated age-related metabolic decline by preserving the expression of metabolism- and muscle contraction-related genes while also potentially reducing chronic inflammation and tumorigenesis through the suppression of inflammation- and tumorigenesis-associated gene expression.

Collectively, our results suggest that NIC could alleviate aging phenotypes, such as frailty, decline in physical function, and metabolic impairment, by suppressing age-associated mTORC1 hyperactivation and improving autophagic flux in aging models.

## Discussion

Autophagy is an essential mechanism for maintaining homeostasis in organisms, and a decline in autophagy activity due to aging leads to functional decline and age-related diseases. Furthermore, as mentioned above, autophagy has been linked to longevity in various organisms. Therefore, we screened for compounds known to regulate autophagy that may delay aging using *C. elegans*, which has a short lifespan of 3–4 weeks, making it a strong animal model for studying changes by natural aging [30] and screening compounds [15]. Our screening identified NIC, which attenuated age-related decline in swimming performance. Therefore, further experiments were conducted to evaluate the impact of NIC on aging.

In *C. elegans*, NIC treatment attenuated the age-related decline in motility and mitochondrial function with longevity. Additionally, the accumulation of auto-fluorescence, which accumulates in the body with age and is a marker of physiologically aged states, was reduced by NIC treatment, indicating that homeostatic dysfunction was ameliorated [31]. Subsequently, we evaluated whether the delayed aging effects of NIC in *C. elegans* were consistent in a mammalian mouse model. As an anthelmintic, the recommended dose of NIC for humans is 2 g/day for 7 days, whereas other clinical trials have been conducted at 1 g/day for 6 months [32]. The LD50 value of NIC in rats has been reported to be 5 g/kg, indicating a relatively high safety threshold [33]. Additionally, a previous lifespan study administering the same antioxidant mixture at different starting time points demonstrated that treatment initiated after 16 months did not produce significant effects, unlike earlier administration [34]. Based on these studies, we selected 12 months of age as the starting point for NIC administration [35]. Furthermore, considering the long-term treatment, the concentration was not increased by equating the body surface area, and the same dose per kilogram was used. The high dose was 30 mg/kg/day in the 0.05 % dietary group and the low dose was 15 mg/kg/day in the 0.025 % dietary group. Acute toxicity was not observed during the 9-month treatment. Moreover, no increase was observed in the serum levels of ALT or AST, indicating hepatotoxicity. Thus, we confirmed that these doses were effective concentrations that did not cause toxicity during long-term administration.

Subsequently, we observed that NIC treatment ameliorated frailty phenotypes caused by aging in mice. Frailty is defined as a physical state that precedes disability or is present alongside it. People with a high frailty index have a greater risk of early mortality, falls, and iatrogenic complications compared to same-age people [36]. Therefore, we evaluated overall frailty using a frailty index composed of 31 signs of clinical deterioration [19]. The degree of appetite loss, which accelerates the frailty state, was evaluated by food intake measurement [37,38], and the degree of disability that may occur as frailty progresses was evaluated by behavioral tests such as treadmill, grip strength, rotarod and y-maze tests.

Based on behavioral tests, we focused on skeletal muscles to investigate the effects of NIC and observed that NIC ameliorated age-related deterioration of skeletal muscles. Next, we conducted an experiment to identify the mechanisms mediating the effects of NIC. mTOR is a protein kinase that regulates cellular metabolism, catabolism, autophagy, and proliferation, and is essential for maintaining cellular homeostasis [39]. However, during aging, upregulated mTORC1 activity contributes to the development of diseases such as cancer and neurodegenerative diseases [40,41]. Furthermore, a mild increase in nutrient signaling to mTORC1 in mice leads to shortened lifespan by approximately 30 % [42]. In addition, mTORC1 hyperactivation occurs in skeletal muscles with aging and leads to sarcopenia and instability at the neuromuscular junction. Conversely, inhibition of mTORC1 hyperactivation with rapamycin ameliorates age-related deterioration of skeletal muscle [43]. We observed that NIC inhibited mTORC1 hyperactivation, similar to rapamycin. Moreover, several previous studies have demonstrated that NIC can inhibit mTORC1 activity under conditions of mTORC1 activation, such as in cancer [27,44,45]. Furthermore, in a model of mTORC1 hyperactivation through TSC knockout, the ubiquitin–proteasome system, which promotes protein degradation, is overactivated in skeletal muscle [21]. Similarly, the expression of genes identified as mTORC1-regulated atrogenes and proteasome activity increased in the old group and were attenuated by NIC treatment. Moreover, in a short-lived nematode model of LET-363/mTOR hyperactivation, we observed that NIC restored the lifespan by inhibiting hyperactivation.

Subsequently, we evaluated the effect of NIC on autophagy induced by the inhibition of mTORC1 signaling. Contrary to our expectations, the expression of LC3, which forms autophagosomes, was lower in the NIC treatment group than that in the old group. However, we also observed that the levels of P62, NBR1, BNIP3, FUNDC1, and Ubiquitin, which label materials to be degraded or directed to the autophagosome, were consistently higher in the old group than in the young group and were reduced by NIC treatment. These results suggest that NIC may promote autophagy by attenuating the impairment of the autophagic flux with aging, and not by increasing the level of autophagy. Observation of autophagic vacuoles by TEM imaging confirmed that the increase in LC3B expression in the old group was due to an increase in autophagic vacuole size. This is consistent with numerous studies that have reported an increase in the size and number of autophagic vacuoles under aging or chronic oxidative stress [[46], [47], [48]].

Thereafter, we performed additional animal experiments using leupeptin, which inhibits autolysosomal degradation to assess the effect of NIC on the autophagic flux more clearly. We observed that the expression of LC3B, which varied with leupeptin treatment, significantly increased in the NIC treatment group compared to that in the vehicle group. We also consistently observed that NIC treatment in *C. elegans* did not increase the expression of LGG-1/LC3B, but rather promoted autophagy by ameliorating age-related impairment of autophagic flux.

We further performed mRNA-seq analysis using skeletal muscle tissue to evaluate the overall impact of NIC and found that numerous genes involved in the contraction and metabolic functions of skeletal muscle were upregulated in both the young and NIC treatment group compared to the old group. Among the analyzed genes, glycerol-3-phosphate dehydrogenase (GDP) 1 and GDP2 function in the regeneration of NAD^+^ and electron transfer to the electron transport chain in the cytosol and mitochondria, respectively. Their reduced expression indicates decreased metabolic function [[49], [50], [51], [52]]. Furthermore, considering the increased expression of genes such as *Agl*, *Pygm*, and *Phka1*, which are involved in the catabolism of glycogen, and *Camk2a*, *Calm3*, *Casq1*, *Srl*, and *Sypl2*, which are involved in the contraction of skeletal muscle, NIC treatment improved metabolic function compared to the old group [[53], [54], [55], [56], [57], [58]]. Moreover, suppression of excess fat accumulation was observed in the NIC treatment group. Additionally, the NIC-treated groups showed improvements in both the age-related deterioration of mitochondrial morphology and function in skeletal muscle, as well as the reduction in energy expenditure. Our results are consistent with previous studies which have demonstrated that NIC can improve obesity [59] and type 2 diabetes [60] by improving metabolic function. Furthermore, NIC treatment-induced changes such as lower body weight, higher dietary intake, and exercise performance were similar to the results of a previous study in which aging mice were treated with rapamycin [43].

These results suggest that NIC treatment alleviates the decline of homeostatic systems, such as autophagy, thereby enabling proper function of metabolic systems, including mitochondria. Consequently, this leads to reduced fat accumulation and a slower decline in exercise capacity.

In conclusion, we demonstrated that NIC ameliorates frailty and age-associated metabolic and physical decline in nematodes and mice. These beneficial effects are mediated through the suppression of age-associated mTORC1 hyperactivation and the subsequent improvement of autophagic flux. However, further studies are necessary to elucidate how mTORC1 hyperactivation occurs in skeletal muscle during aging and how NIC inhibits mTORC1 hyperactivation. Additionally, the evaluation of the efficacy of NIC in tissues other than skeletal muscle remains insufficient. Notably, the inverse correlation between body weight and behavioral performance indicates that suppression of age-related fat accumulation may play a contributing role in maintaining physical performance (Supplementary Fig. 14). Therefore, further studies are required to explore the effects of NIC on hepatic and adipose tissue aging. Despite these limitations, we advocate that NIC is a promising anti-aging agent, similar to rapamycin.

## Declaration of competing interest

The authors declare that they have no known competing financial interests or personal relationships that could have appeared to influence the work reported in this paper.

## Appendix A Supplementary data

Supplementary Data 1

Supplementary video 1 (Niclosamide)

Supplementary video 2 (Vehicle)
