# Supplementary material for: Niclosamide extends health span and reduces frailty by ameliorating mTORC1 hyperactivation in aging models
Source: J Adv Res. 2025 Apr 22;80:1031–44. doi: 10.1016/j.jare.2025.04.027 (PMC12869201; doi:10.1016/j.jare.2025.04.027)
Supplement: Supplementary Data 1 [file mmc1.docx]

**Supplementary materials and methods**

**Body composition measurement**

Body composition was measured using an InAlyzer (Medikors Inc., Gyeonggi-do Republic of Korea), which uses dual-energy X-ray absorptiometry (DEXA). The mice were anesthetized with 2% isoflurane to prevent movement during measurement and scanning. The ratio of fat mass to lean body mass was obtained using the InAlyzer software.

**Histological analysis**

Liver and epididymal (EP) fat were fixed in 4% formaldehyde and embedded in paraffin. The paraffin blocks were sectioned (4 µm), deparaffinized, rehydrated, and strained using hematoxylin and eosin (H&E)-phloxine solution. The quadriceps were placed in 30% sucrose and embedded in Tissue-Tek O.C.T. compound (4583, Sakura Finetek, Osaka, Japan) and frozen at -80 °C. After sectioning at 7 µm, H&E and Oil red O staining were performed. The slides were scanned using a Pannoramic 250 flash 3 scanner (3Dhistech, Budapest, Hungary) and quantified using ImageJ software (National Institutes of Health).

**Triglyceride (TG) content measurement**

TG content in the quadriceps muscle was measured using an EZ-Triglyceride Quantification Assay Kit (DG-TGC100, Dogenbio, Seoul, Republic of Korea). Briefly, TG was broken down into free fatty acids and glycerol. The glycerol was oxidized and the resulting hydrogen peroxide was reacted with the probe. Absorbance was measured using a Synergy H1 microplate reader (Agilent Technologies).

**Energy expenditure analysis**

Energy expenditure was measured using the OxyletPro Physiocage system (Panlab, Barcelona, Spain) and Metabolism V3.0 software (Panlab). The mice were placed in a single metabolic chamber in which oxygen consumption (VO_2_) and carbon dioxide production (VCO_2_) were measured over 24 h. Energy expenditure was calculated automatically by determining the respiratory quotient from the measured VCO_2_ and VO_2_.

**Measurement of serum cytokine and chemokine Levels by ELISA**

Serum concentrations of TNF-α, MCP-1, and IL-6 were measured using ELISA kits (BioLegend, CA, USA) according to the manufacturer’s instructions. Briefly, serum samples were collected and stored at -80°C until analysis. The samples were thawed on ice, and 50 µL of each sample was added to a 96-well ELISA plate pre-coated with capture antibodies specific to TNF-α, MCP-1, and IL-6. Cytokine concentrations were calculated by generating a standard curve using the provided standards and expressed as pg/mL.

**Supplementary Figure 1. Niclosamide does not increase lifespan in mice**


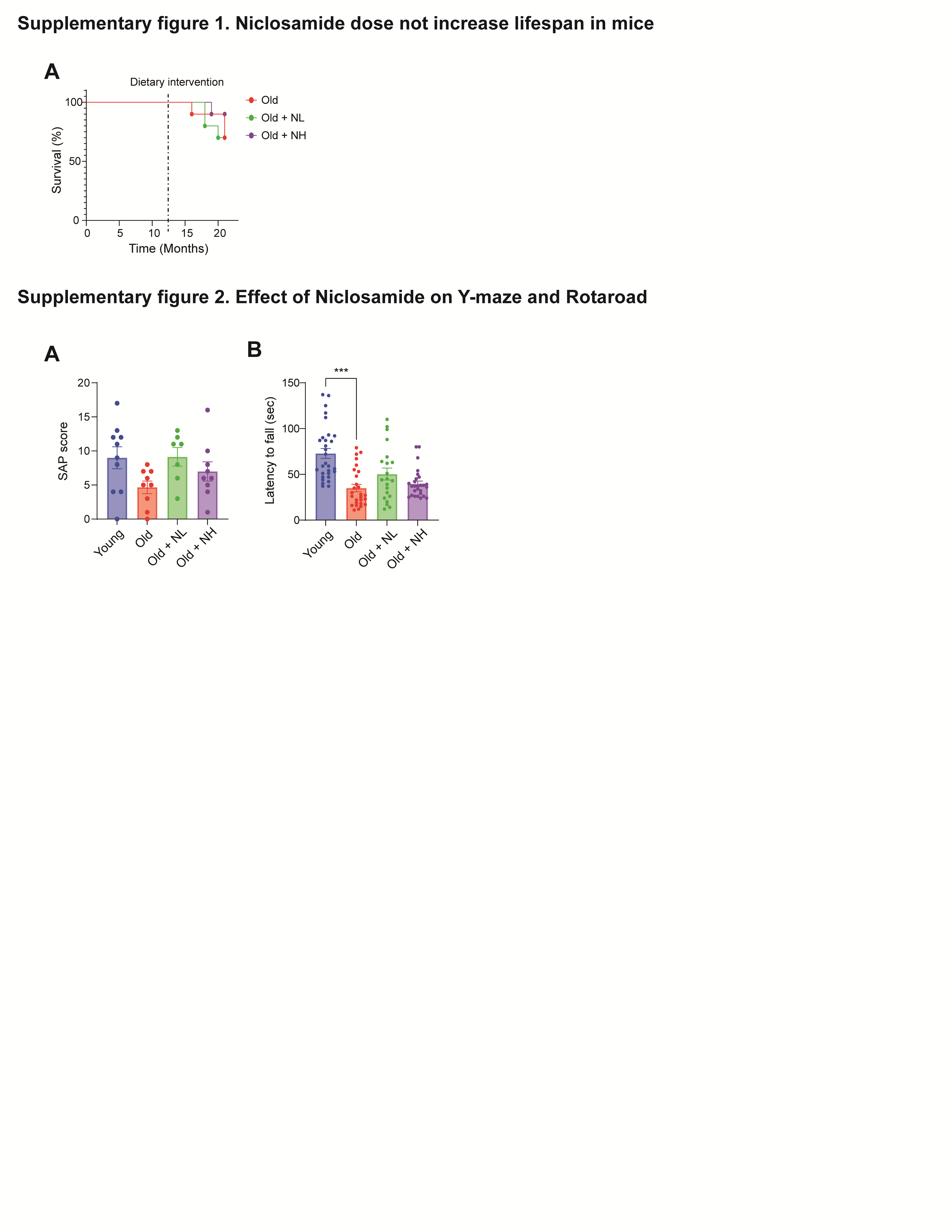


NIC (0%, 0.025%, or 0.05%) was included in the diet and administered for 9 months from 12 to 21 months of age. (**A**) Effect of NIC on Survival rates. NIC: Niclosamide, NL: Niclosamide low dose treatment group (0.025%), NH: Niclosamide high dose treatment group (0.05%).

**Supplementary Figure 2. Niclosamide ameliorates aging-associated excess fat accumulation and increases energy expenditure in mice**


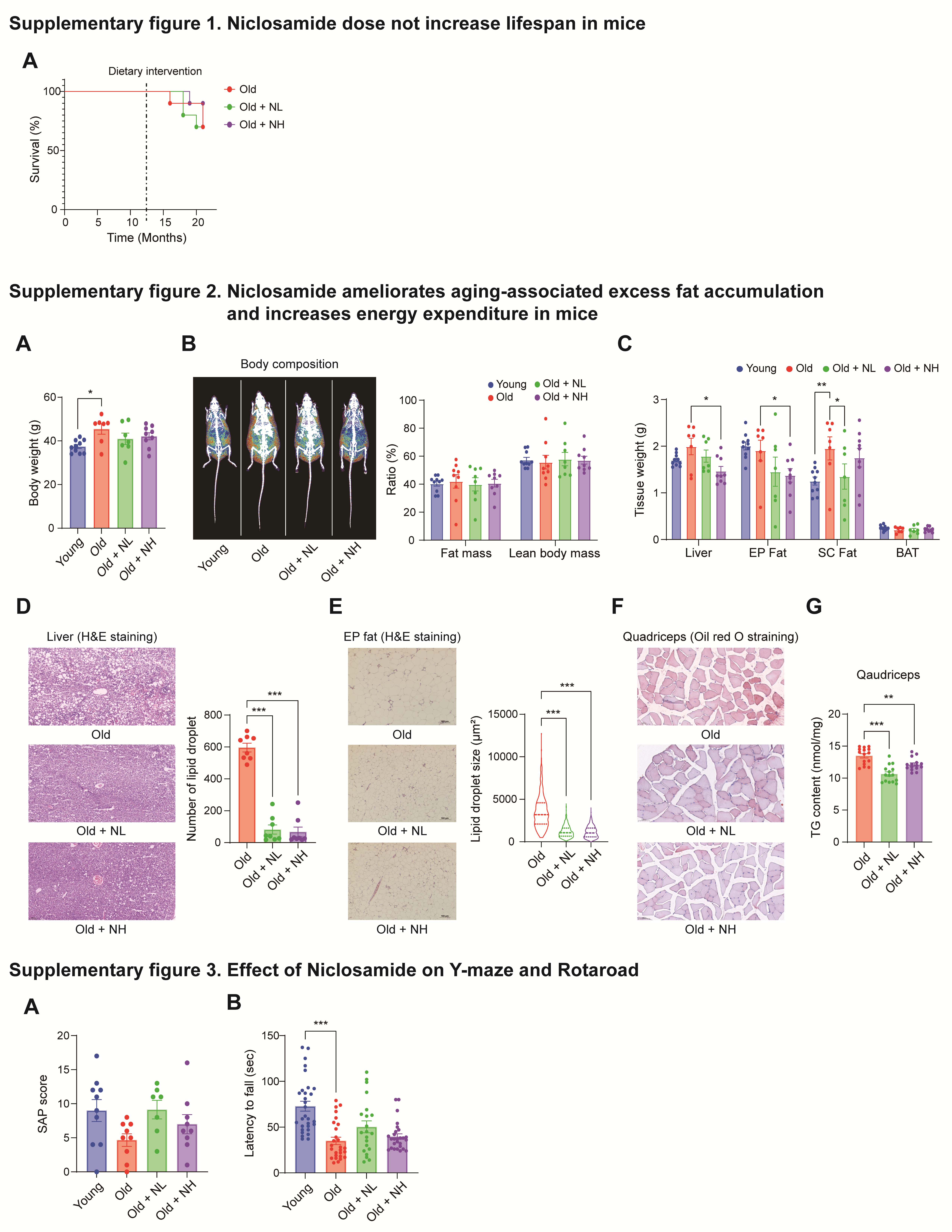


NIC (0%, 0.025%, or 0.05%) was included in the diet and administered for 9 months from 12 to 21 months of age. Six-month-old mice were used as the young group. Effects of NIC on (**A**) Body weight, (**B**) Body composition, and (**C**) Tissue weight (Liver, EP fat, SC fat, and BAT). Histological analysis of (**D**) Liver and (**E**) EP fat using H&E staining (Scale bar: 100 µm). (**F**) Histological analysis of the quadriceps muscles using Oil Red O staining (scale bar: 50 µm). (**G**) Effect of NIC on TG content in quadriceps muscles. NIC: Niclosamide, NL: Niclosamide low-dose treated group (0.025%), NH: Niclosamide high-dose treated group (0.05%), EP fat: Epididymal fat tissue, SC fat: Subcutaneous fat tissue, BAT: Brown adipose tissue, H&E staining: Hematoxylin and eosin staining, TG: Triglyceride, * p < 0.05, ** p < 0.01, *** p < 0.001.

**Supplementary Figure 3. Effect of niclosamide on Y-maze and Rotaroad experiments**


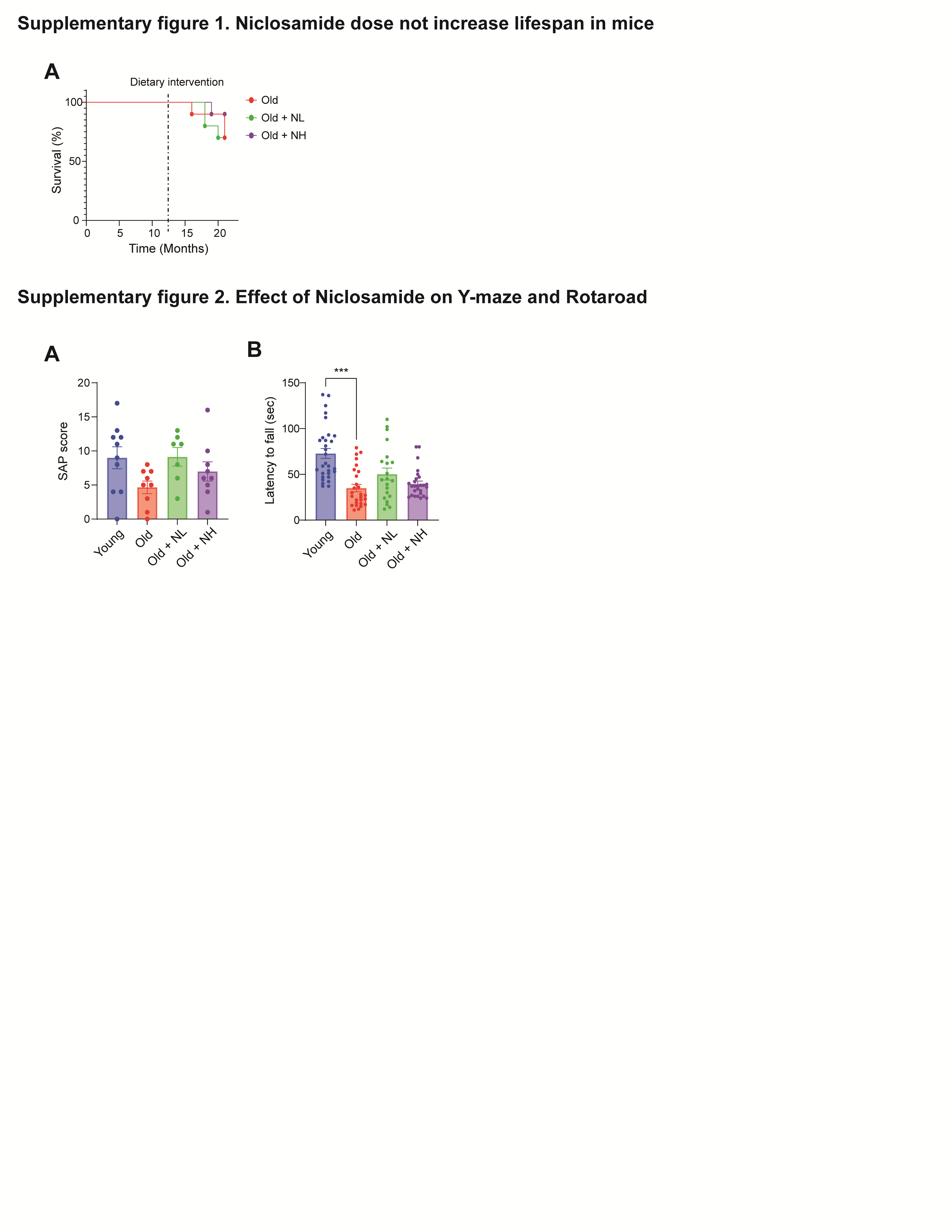


NIC (0%, 0.025%, or 0.05%) was included in the diet and administered for 9 months from 12 to 21 months of age. 6-month-old mice were used the young group. (**A**) Effect of NIC on SAP scores in the Y-maze test. (**B**) The effect of NIC on latency of falls in the rotaroad test. NIC: Niclosamide, NL: Niclosamide low-dose treatment group (0.025%), NH: Niclosamide high-dose treatment group (0.05%), SAP: Spontaneous alternation performance, *** p < 0.001.

**Supplementary Figure 4. Gene expression level of lipogenesis-related genes in liver and EP fat**


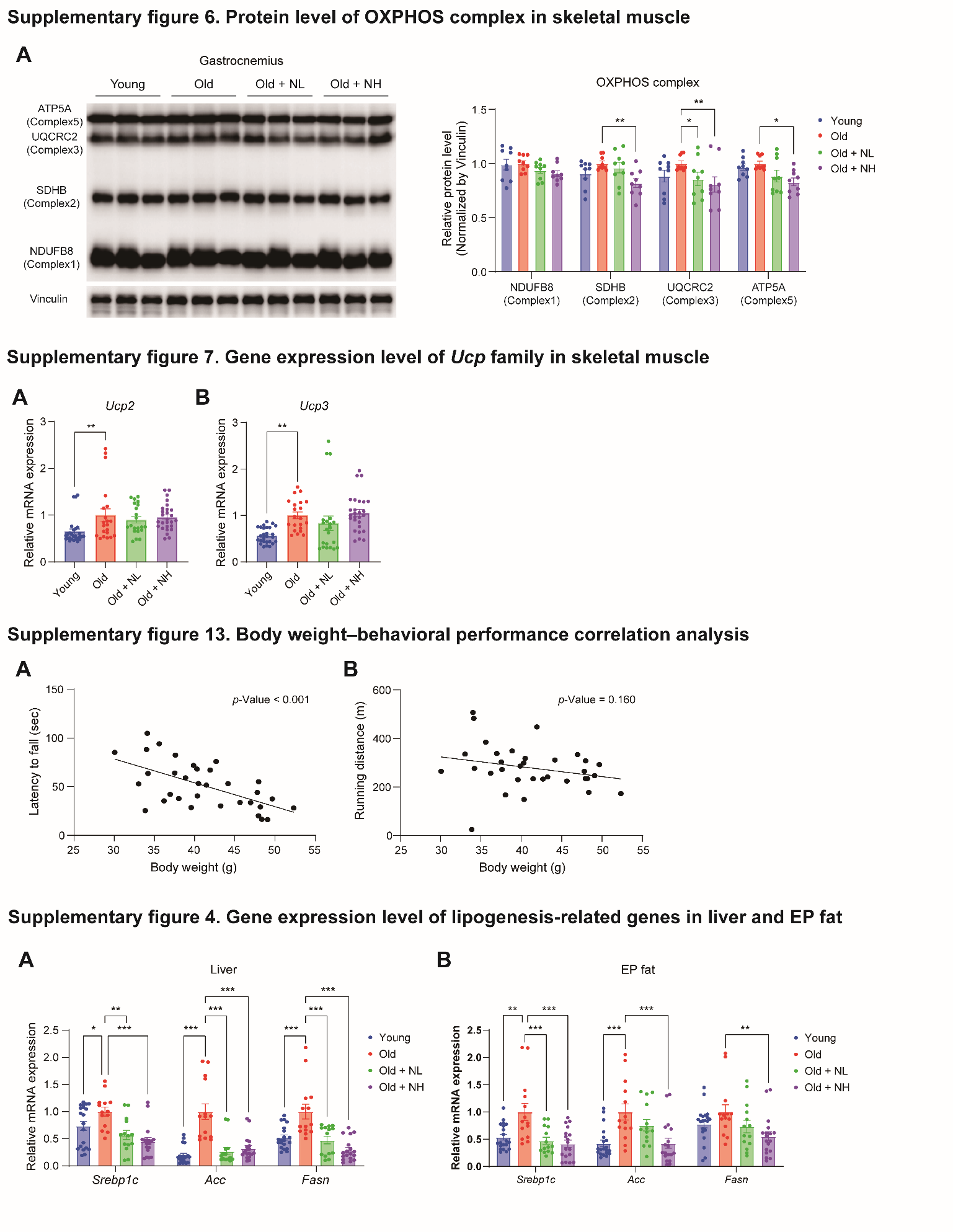


NIC (0%, 0.025%, or 0.05%) was included in the diet and administered for 9 months from 12 – 21 months of age. Six-month-old mice were used as the young group. Lipogenesis-related genes expression level in (**A**) Liver and (**B**) EP fat. NL: Niclosamide low dose treated group (0.025%), NH: Niclosamide high dose treated group (0.05%), EP fat: Epididymal fat tissue, * p < 0.05, ** p < 0.01, *** p < 0.001.

**Supplementary Figure 5. Protein level of aging marker in liver and skeletal muscle**


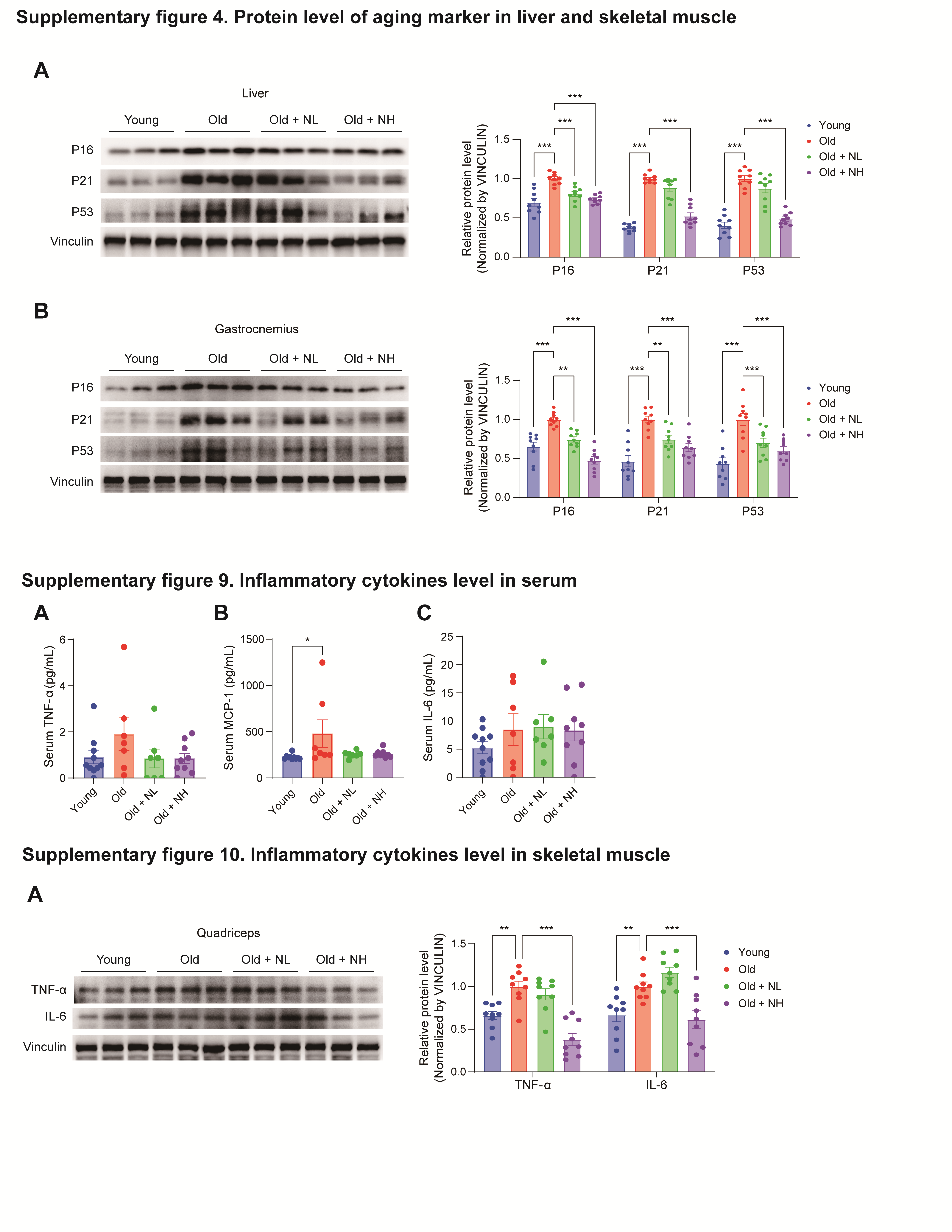


NIC (0%, 0.025%, or 0.05%) was included in the diet and administered for 9 months from 12 – 21 months of age. Six-month-old mice were used as the young group. Western blot analysis of aging markers in (**A**) Liver and (**B**) Gastrocnemius muscle tissues. NL: Niclosamide low dose treated group (0.025%), NH: Niclosamide high dose treated group (0.05%), ** p < 0.01, *** p < 0.001.

**Supplementary Figure 6. Protein level of OXPHOS complex in skeletal muscle**


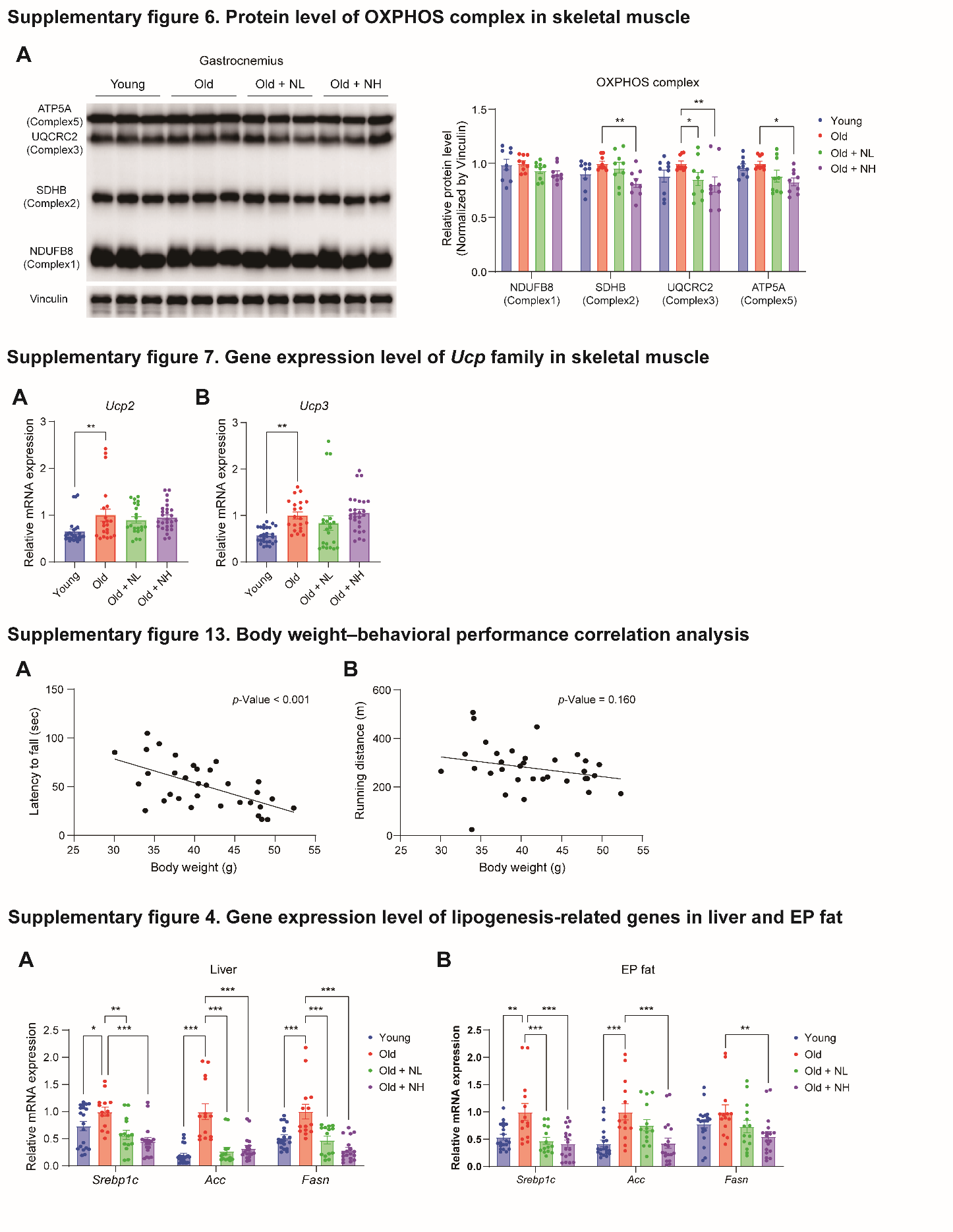


NIC (0%, 0.025%, or 0.05%) was included in the diet and administered for 9 months from 12 – 21 months of age. Six-month-old mice were used as the young group. (**A**) Western blot analysis of OXPHOS complex proteins in gastrocnemius muscle tissues. NL: Niclosamide low dose treated group (0.025%), NH: Niclosamide high dose treated group (0.05%), OXPHOS: Oxidative phosphorylation, * p < 0.05, ** p < 0.01, *** p < 0.001.

**Supplementary Figure 7. Gene expression level of *Ucp* family in skeletal muscle**


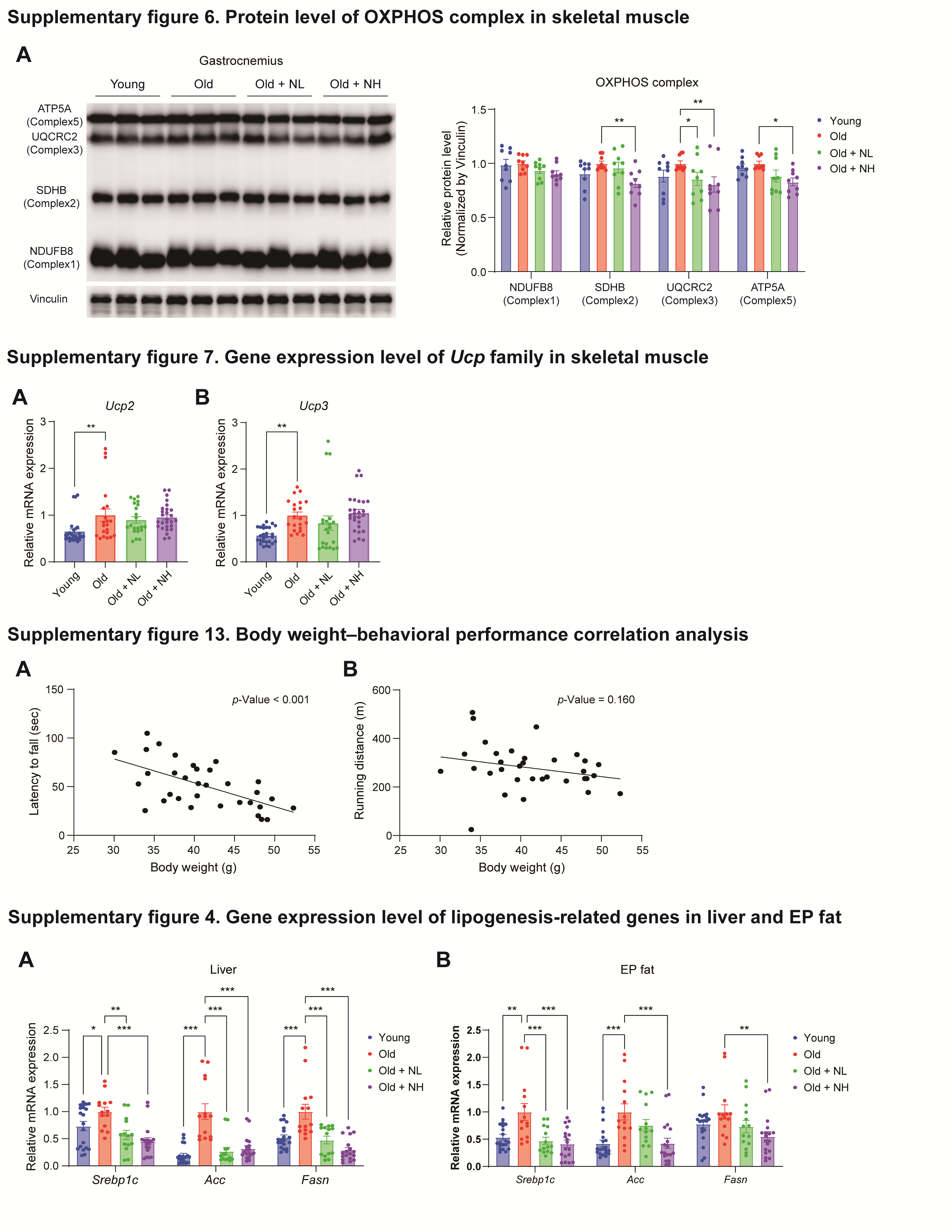


NIC (0%, 0.025%, or 0.05%) was included in the diet and administered for 9 months from 12 – 21 months of age. Six-month-old mice were used as the young group. Gene expression level of (**A**) *Ucp2* and (**B**) *Ucp3* in gastrocnemius muscle tissues. NL: Niclosamide low dose treated group (0.025%), NH: Niclosamide high dose treated group (0.05%), ** p < 0.01.

**Supplementary Figure 8. Effect of niclosamide on autophagic vacuoles in skeletal muscle**


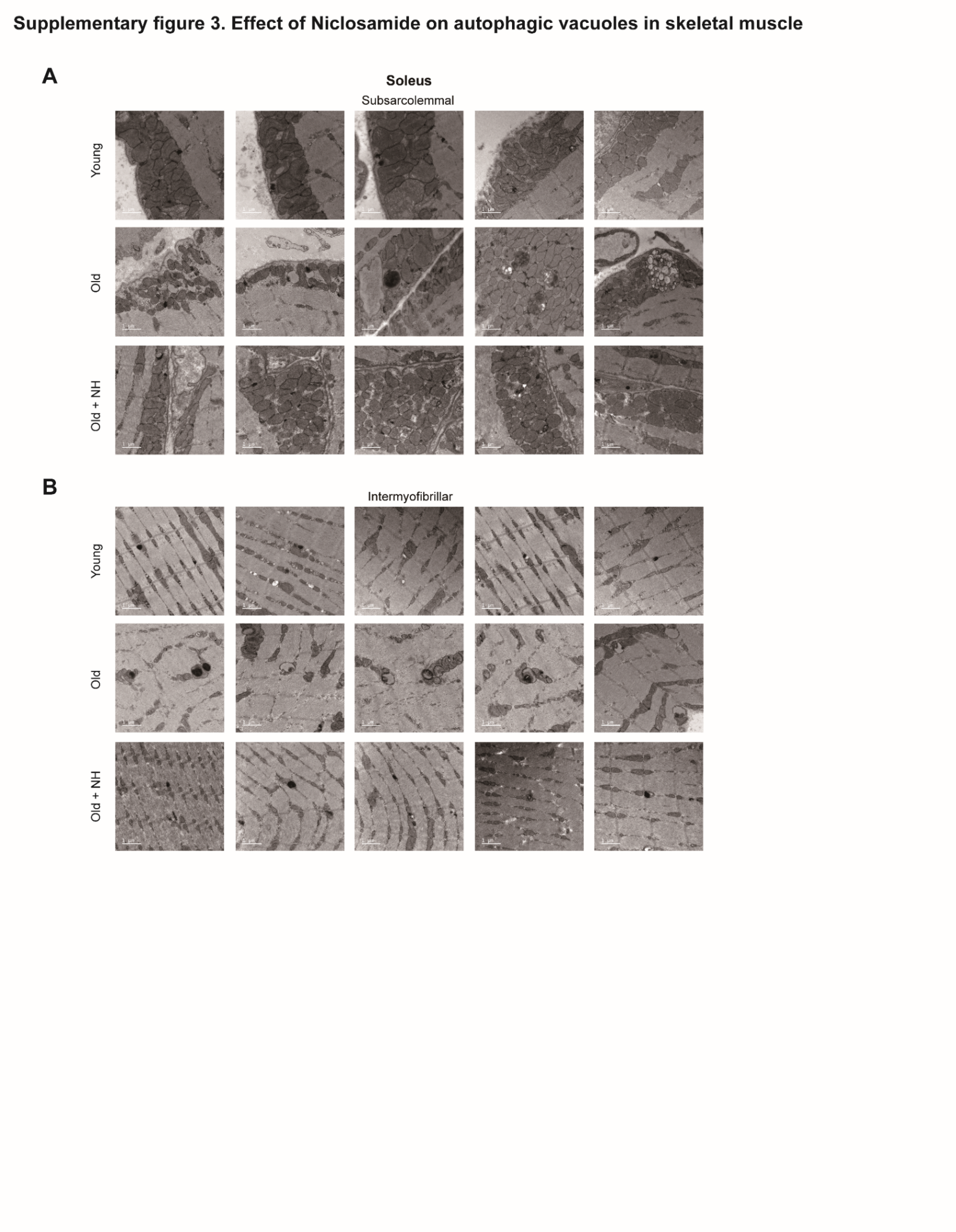


NIC (0% or 0.05%) was included in the diet and administered for 9 months from 12 to 21 months of age. Six-month-old mice were used as the young group. Effect of NIC on the morphology of autophagic vacuoles by TEM imaging in the soleus muscle (scale bar: 1 µm). (**A**) Subsarcolemmal area image. (**B**) Intermyofibrillary area images. NIC: Niclosamide, NH: Niclosamide high-dose treatment group (0.05%), TEM: Transmission electron microscopy.

**Supplementary Figure 9. Effect of niclosamide on middle-aged mice**


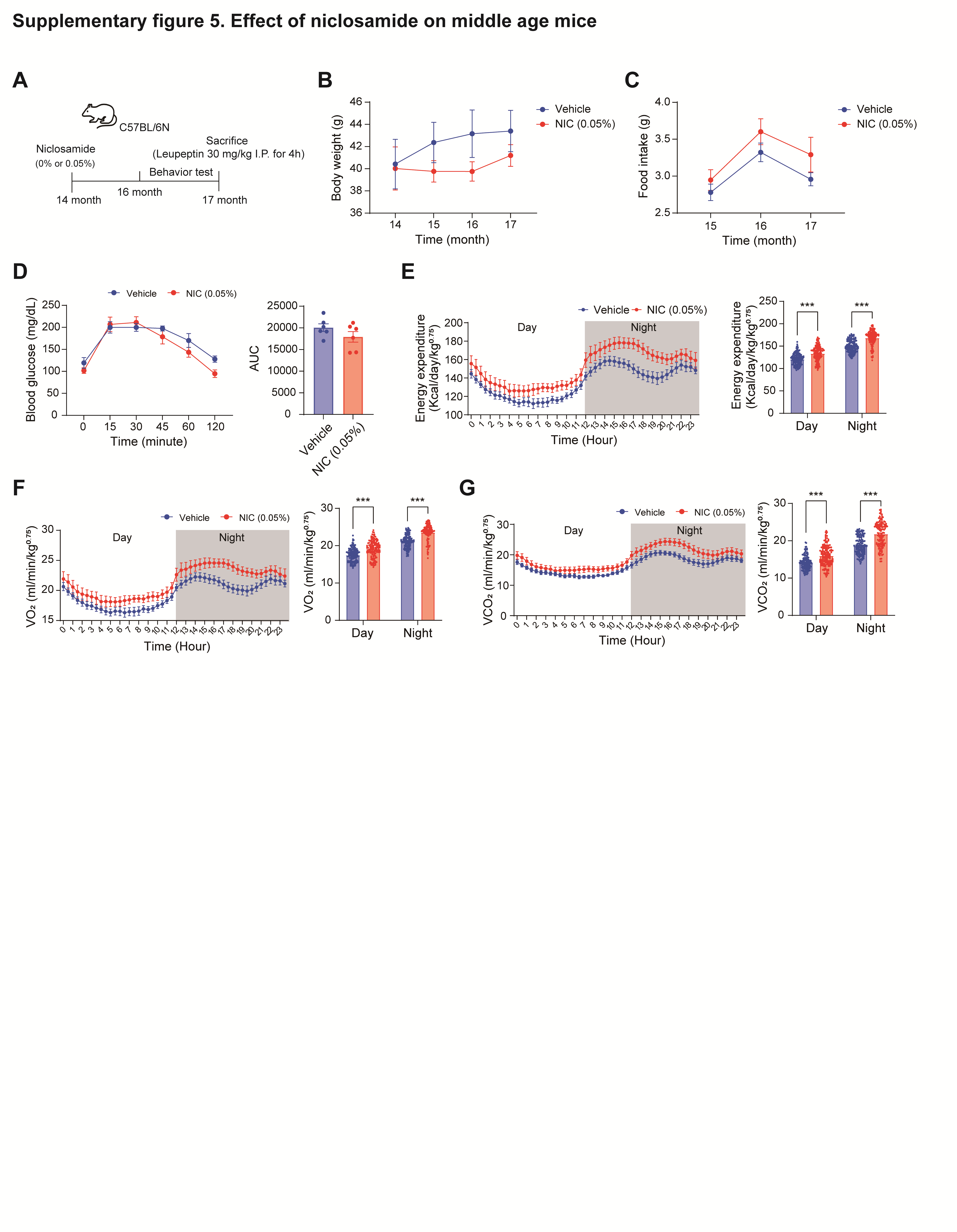


NIC (0% or 0.05%) was included in the diet and administered for 3 months, from 14 to 17 months of age. (**A**) Experimental design. Effects of NIC on (**B**) Body weight and (**C**) Food intake. (**D**) Effect of NIC on blood glucose levels measured by OGTT. Effect of NIC on (**E**) Energy expenditure, (**F**) VO_2_ and (**G**) VCO_2_. NIC: Niclosamide, NIC (0.05%): Niclosamide high-dose treatment group (0.05%), AUC: Area under the curve, OGTT: Oral glucose tolerance test, *** p < 0.001.

**Supplementary Figure 10. Specific signaling pathway related-gene expression profile in niclosamide treated quadriceps muscles**


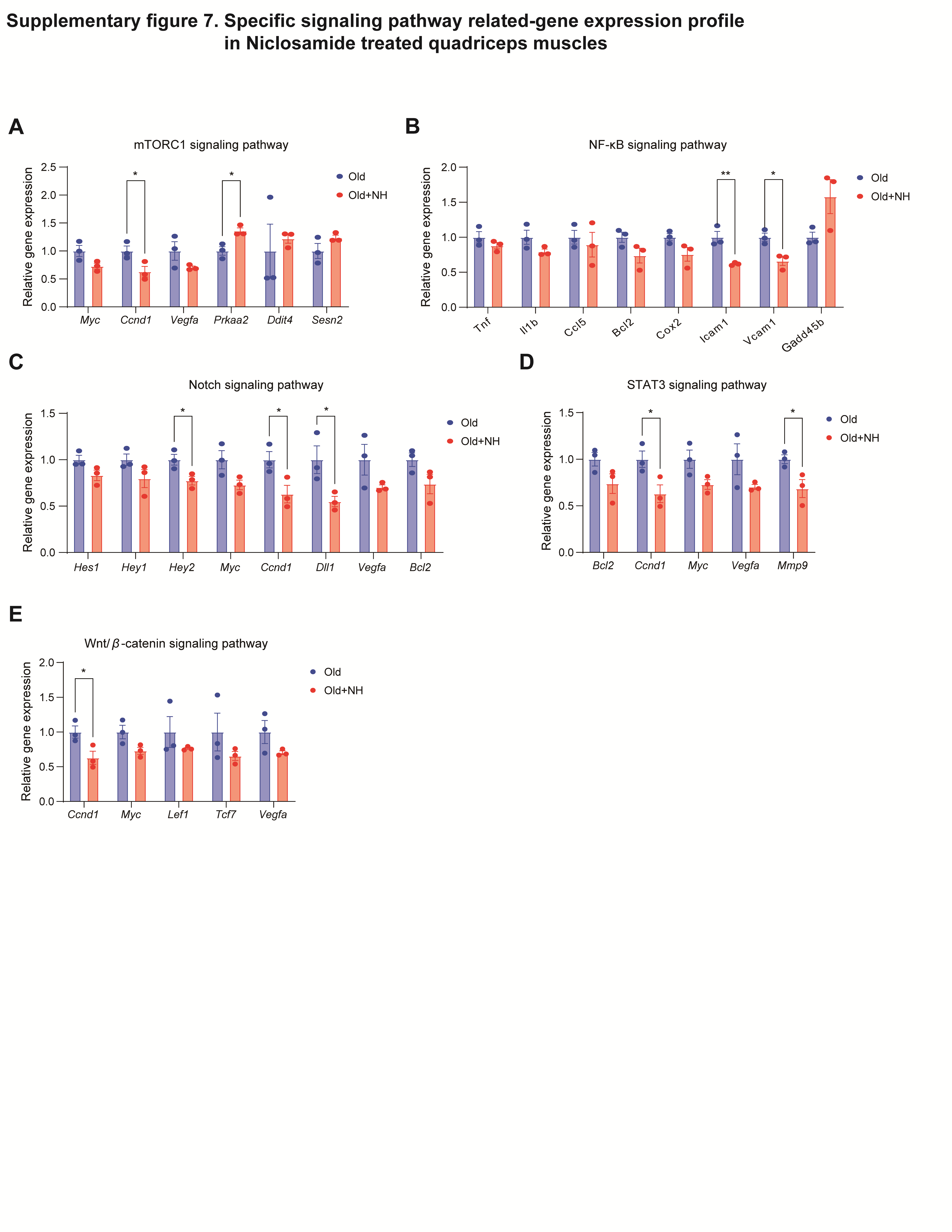


NIC (0% or 0.05%) was added to the diet and administered for 9 months from 12 – 21 months of age. Gene expression profiles in quadriceps muscles were analyzed. The expression of genes related (**A**) mTORC1 signaling pathway, (**B**) NF-κB signaling pathway, (**C**) Notch signaling pathway, (**D**) STAT3 signaling pathway, (**E**) Wnt/β-catenin signaling pathway. NH: Niclosamide high dose treated group (0.05%), * p < 0.05, ** p < 0.01.

**Supplementary Figure 11. KEGG pathway and GO biological process analysis in niclosamide treated quadriceps muscles**


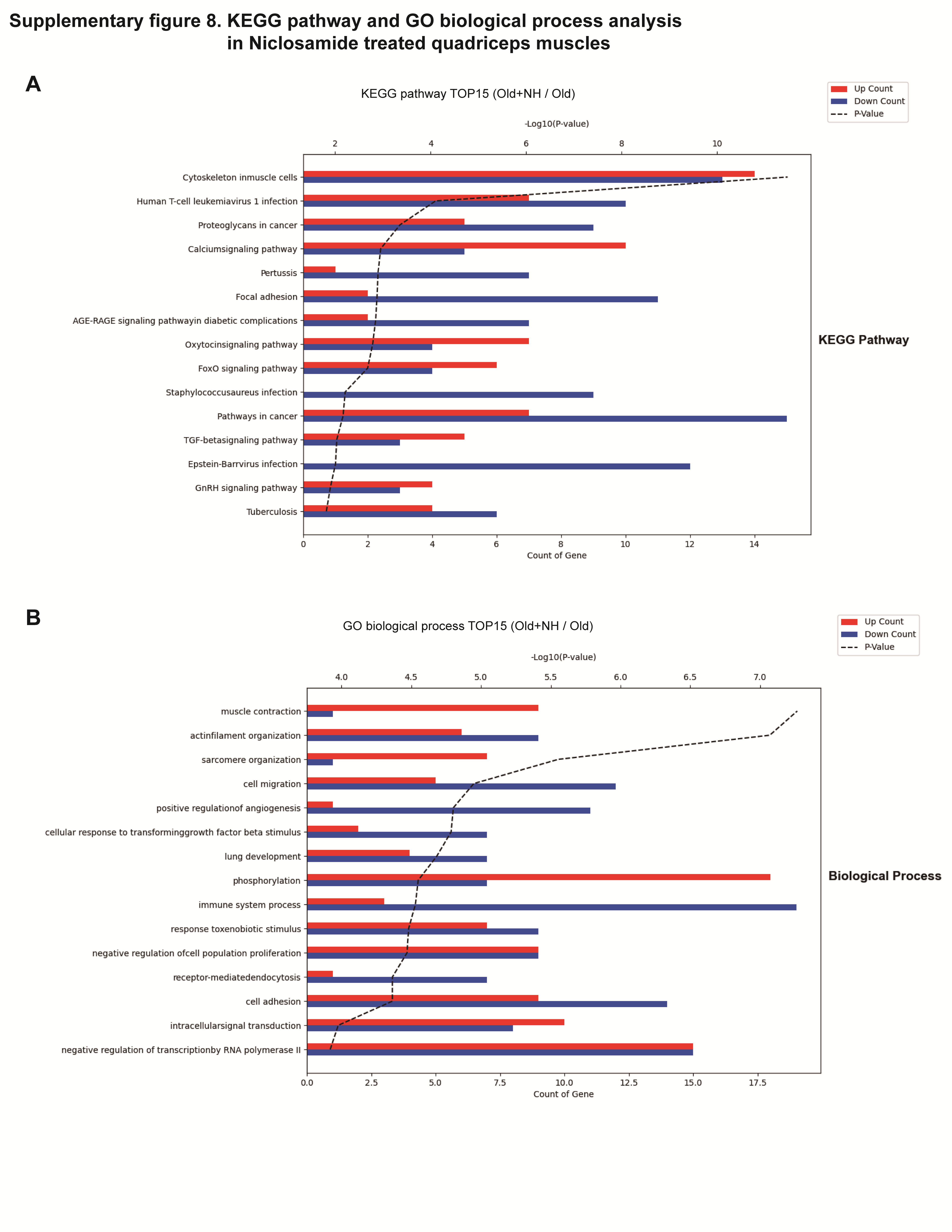


NIC (0% or 0.05%) was added to the diet and administered for 9 months from 12 – 21 months of age. Gene expression profiles in quadriceps muscles were analyzed. d (**A**) KEGG pathway analysis. (**B**) GO biological process analysis. NH: Niclosamide high dose treated group (0.05%).

**Supplementary Figure 12. Inflammatory cytokines and chemokine level in serum**


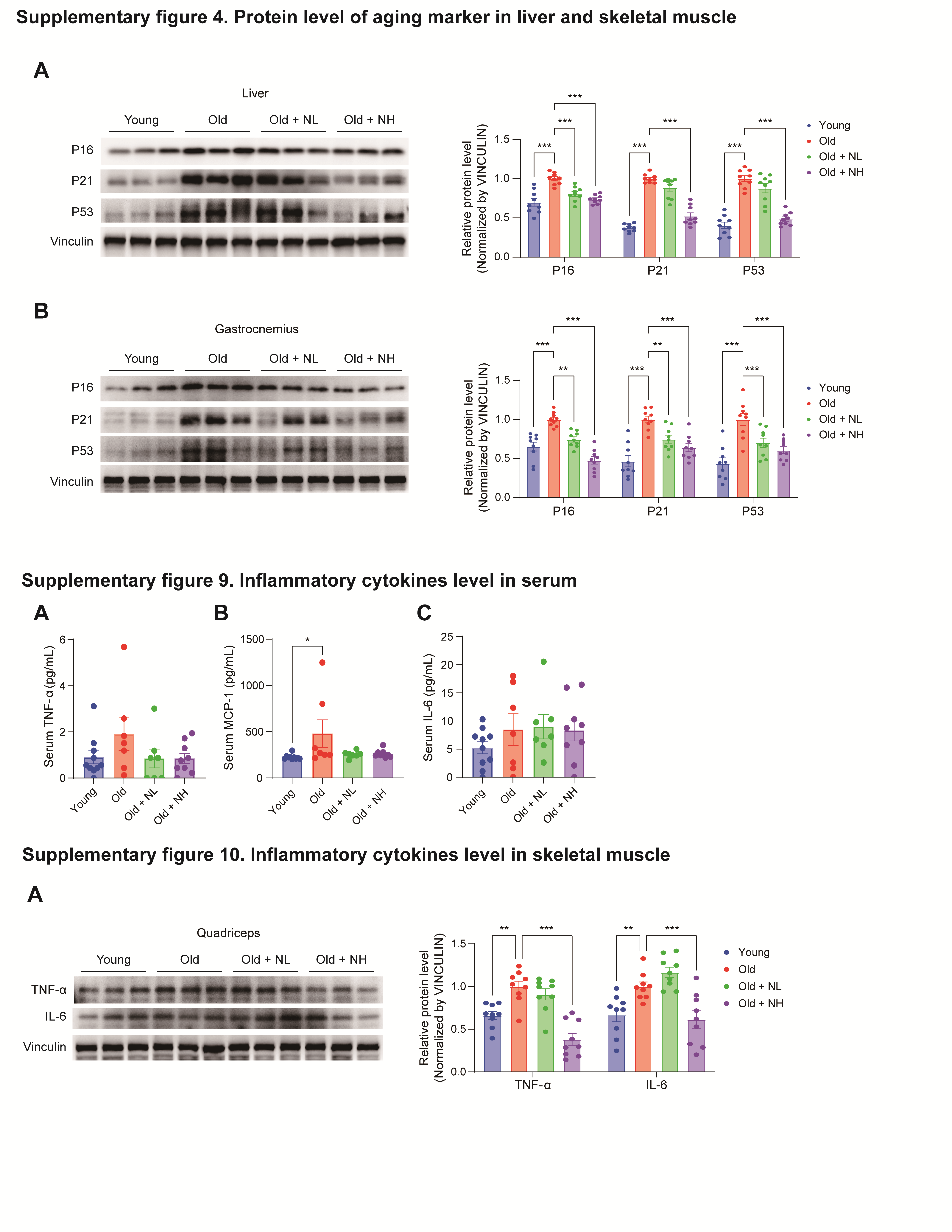


NIC (0%, 0.025%, or 0.05%) was included in the diet and administered for 9 months from 12 – 21 months of age. Six-month-old mice were used as the young group. The level of Inflammatory cytokine and chemokine in the serum (**A**) TNF-α, (**B**) MCP-1, and (**C**) IL-6. NL: Niclosamide low dose treated group (0.025%), NH: Niclosamide high dose treated group (0.05%), * p < 0.05.

**Supplementary Figure 13. Inflammatory cytokines level in skeletal muscle**


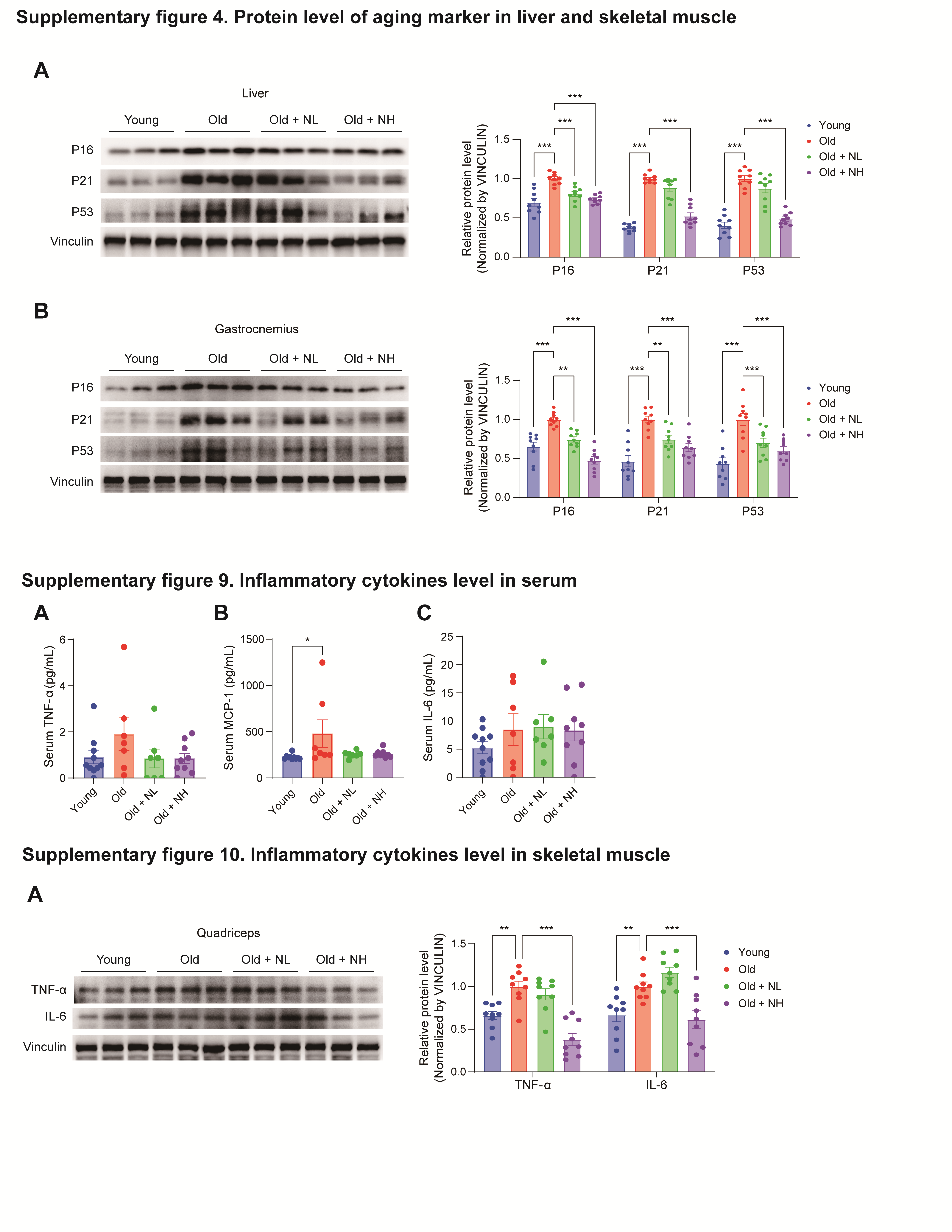


NIC (0%, 0.025%, or 0.05%) was included in the diet and administered for 9 months from 12 – 21 months of age. Six-month-old mice were used as the young group. (**A**) Western blot analysis of inflammatory cytokine in quadriceps muscle tissues. NL: Niclosamide low dose treated group (0.025%), NH: Niclosamide high dose treated group (0.05%), ** p < 0.01, *** p < 0.001.

**Supplementary Figure 14. Body weight-Behavioral performance correlation analysis**

**
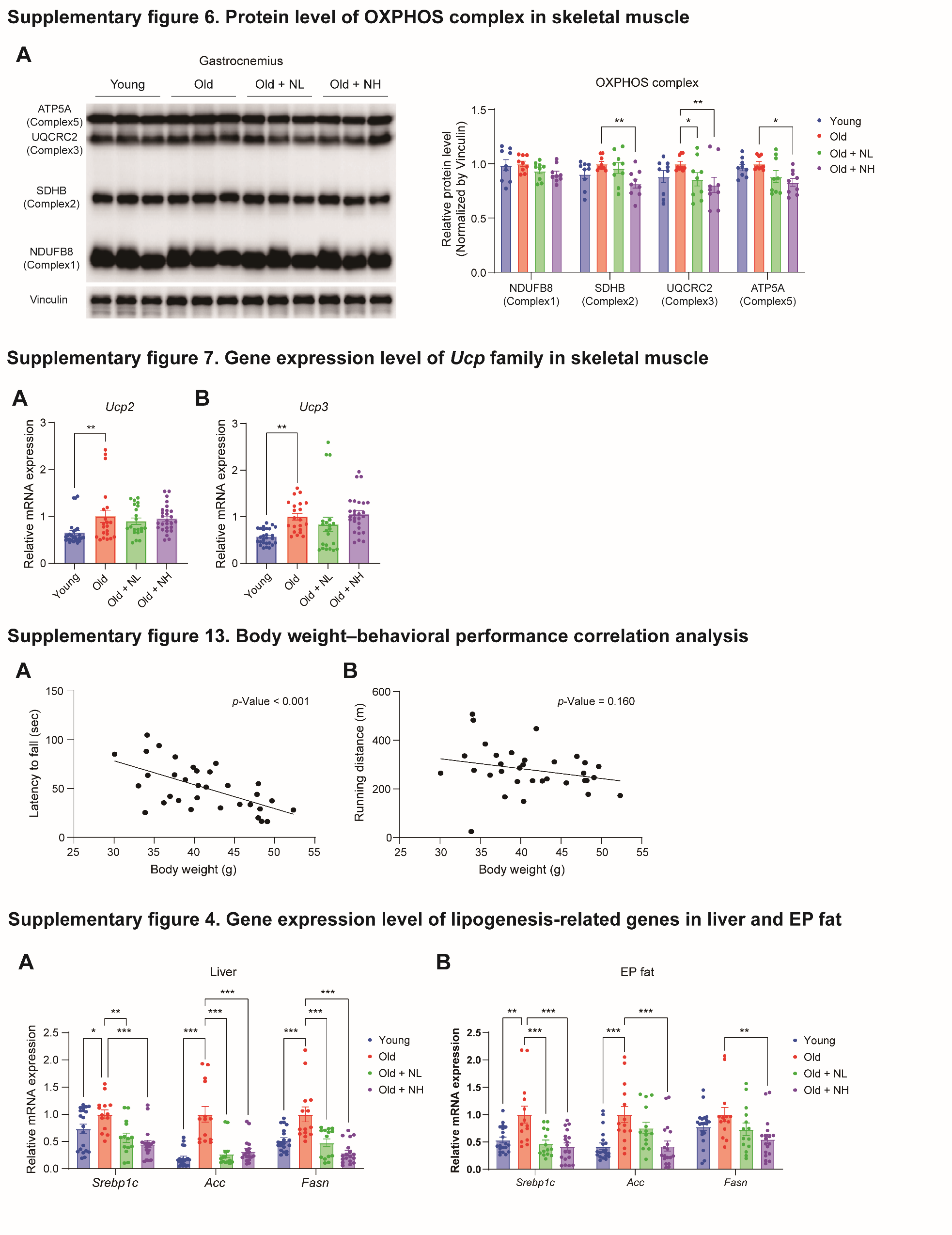
**

NIC (0%, 0.025%, or 0.05%) was included in the diet and administered for 9 months from 12 to 21 months of age. Six-month-old mice were used as the young group. (**A**) Correlation analysis between body weight and latency to fall, assessed using rotarod test correlation analysis. (**B**) Correlation analysis between body weight and running distance, measured using treadmill test.

**Supplementary Table 1. Anti-body list**

| **Target protein** | **Company, Country** | **Catalog number** |
| --- | --- | --- |
| **Total-MHC** | DSHB, USA | MF-20 |
| **Vinculin** | Sigma-Aldrich, USA | V9131 |
| **Phospho-mTOR (Ser2448)** | Cell Signaling, USA | 5536 |
| **mTOR** | Cell Signaling, USA | 2983 |
| **Phospho-p70 S6 Kinase (Thr389)** | Cell Signaling, USA | 9205 |
| **p70 S6 Kinase** | Cell Signaling, USA | 2708 |
| **Phospho-ULK1 (Ser757)** | Cell Signaling, USA | 6888S |
| **ULK1** | Cell Signaling, USA | 8054 |
| **Ubiquitin** | Cell Signaling, USA | 91112 |
| **P62** | MBL Life science, Japan | PM045 |
| **NBR1** | Proteintech, USA | 16004-1-AP |
| **Total OXPHOS cocktail** | Abcam, USA | ab110413 |
| **BNIP3** | Abcam, USA | ab109362 |
| **FUNDC1** | Abcam, USA | ab74834 |
| **TNF-α** | Abcam, USA | ab66579 |
| **GFP** | Santa cruz, USA | sc-9996 |
| **ACTIN** | Santa cruz, USA | sc-47778 |
| **IL-6** | Santa cruz, USA | sc-1265 |
| **P16** | Santa cruz, USA | sc-166760 |
| **P21** | Santa cruz, USA | sc-6246 |
| **P53** | Santa cruz, USA | sc-126 |
| **LC3B** | Novus, USA | NB100-2220 |
| **goat anti-rabbit IgG-HRP** | Novus, USA | NB7160 |
| **goat anti-mouse IgG-HRP** | Novus, USA | NB7539 |

**Supplementary Table 2. List of primers used in mice experiments**

| Gene | Direction | Primer sequences (5’-3’) |
| --- | --- | --- |
| *Fbxo32* | Forward | GACTGGACTTCTCGACTGCC |
|  | Reverse | TCAGGGATGTGAGCTGTGAC |
| *Trim63* | Forward | GCTGGTGGAAAACATCATTGACAT |
|  | Reverse | CATCGGGTGGCTGCCTTT |
| *Fbxo30* | Forward | TCGTGGAATGGTAATCTTGC |
|  | Reverse | CCTCCGTTTCTCTATCACG |
| *Mdm2* | Forward | GAGGATGATGAGGTCTATCG |
|  | Reverse | GGAGGATTCATTTCATTGCAC |
| *Psmc1* | Forward | AAGGGGGTCATTCTCTACGG |
|  | Reverse | AAGCTCTGAGCCAACCACTC |
| *Psmd4* | Forward | TCTCCTATTCTGGCTGGTGAA |
|  | Reverse | CATGCTGCTTAGGTCTGGAAG |
| *Psmd8* | Forward | GCCTCAATCTCCTCTTCCTGCTATC |
|  | Reverse | GTCTGTCATCTTTTTGGGTGTGC |
| *Psme4* | Forward | AGCGTCAACAAGATAAGAATGCT |
|  | Reverse | GCCCGATTCCTATATGCTCAAA |
| *18s* | Forward | CTCAACACGGGAAACCTCAC |
|  | Reverse | CGCTCCACCAACTAAGAACG |
| *Srebp1c* | Forward | GGAGCCATGGATTGCACATT |
|  | Reverse | GGCCCGGGAAGTCACTGT |
| *Acc* | Forward | TGACAGACTGATCGCAGAGAAAG |
|  | Reverse | TGGAGAGCCCCACACACA |
| *Fasn* | Forward | GCTGCGGAAACTTCAGGAAAT |
|  | Reverse | AGAGACGTGTCACTCCTGGACTT |

**Supplementary Table 3. List of primers used in *C. elegans* experiments**

| Gene | Direction | Primer sequences (5’-3’) |
| --- | --- | --- |
| *lgg-1* | Forward | CAACTCTACCAGGACCATCAC |
|  | Reverse | ACAAGTATACACATTCGTCGG |
| *sqst-1* | Forward | TCTATACAAGTCACAATCCGCA |
|  | Reverse | CGTTGACATCACCGTAGTAGAG |
| *let-363* | Forward | GCCGATAGACAGAACAAAGCA |
|  | Reverse | CAGCATCAATCCCTTGTTTACGA |
| *ins-7* | Forward | CATGCGAATCGAATACTGAA |
|  | Reverse | CACTGTTTTCGAATGAAGTC |
| *act-1* | Forward | GAATCCACGAGACTTCTTACAAC |
|  | Reverse | GAAGCACTTGCGGTGAACG |
